# Preclinical evidence and therapeutic perspectives on carnosine for the treatment of neurodegenerative disorders

**DOI:** 10.3934/Neuroscience.2025025

**Published:** 2025-10-21

**Authors:** Saviana Antonella Barbati, Giuseppe Carota, Konstantinos Partsinevelos, Lucia Di Pietro, Anna Privitera, Vincenzo Cardaci, Andrea Graziani, Renata Mangione, Giuseppe Lazzarino, Barbara Tavazzi, Valentina Di Pietro, Emiliano Maiani, Francesco Bellia, Angela Maria Amorini, Giacomo Lazzarino, Shahid Pervez Baba, Giuseppe Caruso

**Affiliations:** 1 Departmental Faculty of Medicine, UniCamillus—Saint Camillus International University of Health Sciences, Rome, Italy; 2 IRCCS San Camillo Hospital, Venice, Italy; 3 Department of Biomedical and Biotechnological Sciences, University of Catania, Catania, Italy; 4 Department of Biological, Geological and Environmental Science, University of Catania, Catania, Italy; 5 Università Vita-Salute San Raffaele, Milano, Italy; 6 LTA-Biotech srl, Paternò, Catania, Italy; 7 School of Infection, Inflammation & Immunology, Department of Inflammation and Ageing, College of Medicine and Health, University of Birmingham, Birmingham, United Kingdom; 8 Center for Cardiometabolic Science, Louisville, KY, USA; 9 Department of Medicine, Christina Lee Brown Envirome Institute, University of Louisville, Louisville, KY, USA

**Keywords:** carnosine, *in vivo* studies, animal models, Parkinson's disease, Alzheimer's disease, stroke, cellular and molecular mechanisms, carnosinemia

## Abstract

Carnosine (β-alanyl-L-histidine) is an endogenous dipeptide widely distributed in mammalian tissues, especially skeletal and cardiac muscle cells, and, to a lesser extent, in the brain. While early interest in carnosine was given because of its role in muscle cell metabolism and athletic performance, it has more recently gained attention for its potential application in several chronic diseases. Specifically, brain aging and neurodegenerative disorders have received particular attention, as a marked reduction in carnosine levels has been described in these conditions. Carnosine exerts a wide range of biological activities, including antioxidant, anti-inflammatory, anti-glycation, metal-chelating, and neuroprotective properties. Mechanistically, it acts by inhibiting the production of advanced glycation end products (AGEs), buffering cellular pH, and regulating intracellular nitric oxide signaling and mitochondrial function. Its safety profile, the lack of toxicity, and significant side effects support its application for long-term therapeutic use. In this review, we aim to recapitulate and discuss the effects, dosages, and administration routes of carnosine in preclinical *in vivo* models, with a particular focus on neurodegenerative disorders where it has been shown to reduce oxidative stress, suppress neuroinflammation, modulate protein aggregation, and preserve cognitive function, all key features of neurodegeneration. Despite promising findings, there are gaps in the knowledge on how carnosine affects synaptic plasticity, neuronal remodeling, and other processes that play a central role in the pathophysiology of neurodegenerative disorders. Additionally, clinical translation remains challenging due to inconsistencies across *in vivo* studies in terms of dosage, treatment duration, routes of administration, and disease models, which affect reproducibility and cross-study comparability. Therefore, while carnosine emerges as a multifunctional and well-tolerated molecule, further research is needed to clarify its therapeutic relevance in human diseases. In this review, we also address future perspectives and key methodological challenges that must be overcome to effectively translate carnosine's biological potential into clinical practice.

## 1. Introduction

**Figure 1.**
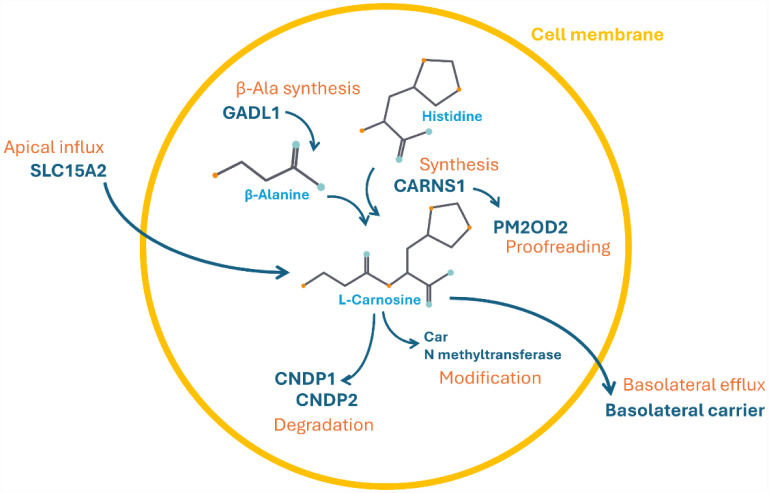
Enzymes regulating carnosine metabolism. Carnosine is synthesized by carnosine synthase 1 (CARNS1) from histidine and β-alanine, the latter being produced by glutamic acid decarboxylase-like 1 (GADL1). Misincorporated products from CARNS1 are metabolized by the peptidase PM20D2. Carnosine degradation is carried out by two dipeptidases: CNDP1 and CNDP2. The methylation of carnosine to form anserine is catalyzed by carnosine N-methyltransferase. Carnosine transport is mediated by two transporters: The apical SLC15A2 and a basolateral carrier.

Carnosine (β-alanyl-L-histidine) is an endogenous dipeptide first isolated in 1900 by Gulewitsch and Amiradzibi, who identified it in meat extracts and named it carnosine from the Latin *caro*, *carnis*, meaning “meat” [1]. Carnosine can be found throughout mammalian tissues, along with its analogs homocarnosine, anserine, and ophidine/balenine [2],[3]. It is particularly abundant in skeletal and cardiac muscles (up to ~20 mM), while a lower but still significant concentration (millimolar order) is present in the brain [4]. Carnosine metabolism involves several enzymes that are responsible for its synthesis, modification, degradation, and transmembrane transport [3],[5] (Figure 1).

Carnosine is synthesized by carnosine synthase 1 (CARNS1) from the amino acids β-alanine (β-Ala) and L-histidine (L-His), both of which are available in the bloodstream. Of the two, β-Ala is produced in the liver, primarily through the degradation of uracil and thymine, or obtained from the diet, while L-His is an essential amino acid and must be provided entirely through dietary intake. Carnosine levels in human tissues and biological fluids are tightly regulated by two carnosinases, enzymes belonging to the M20 metalloprotease family, which catalyze its hydrolysis into β-Ala and L-His. These include the serum enzyme carnosine dipeptidase 1 (CNDP1) and the cytosolic/tissue enzyme carnosine dipeptidase 2 (CNDP2) [6]–[8]. Carnosine exerts a wide range of pleiotropic biological effects, acting not only as a neurotransmitter and cryoprotector in the nervous tissues, particularly in the olfactory bulb and cortex [9], but also as a molecule that regulate immune responses, cellular metabolism, and nitric oxide (NO) signaling [4]. In particular, carnosine is not considered a classic mediator; in fact, in the central olfactory structures it functions as a co-mediator, being in pre-synapses together with the excitatory mediator glutamate. Carnosine multimodal mechanism of action is also characterized by antioxidant [10], anti-inflammatory [11],[12], and anti-aggregating [13] activities along with its ability to chelate heavy metals [14] and sequester reactive aldehydes, such as 4-hydroxynonenal and acrolein [15]. Initially, carnosine studies were mainly focused on skeletal muscle, where supplementation, particularly with carnosine or its precursor β-alanine, was shown to enhance athletic performance by improving muscle contractility and buffering lactic acidosis during intense physical activity. However, it soon became of great interest due to its additionally promising beneficial effects for human health [16],[17]. One of the major reasons behind the growing scientific interest in this dipeptide lies in its excellent safety profile. Both preclinical and clinical studies have confirmed that carnosine is non-toxic, well-tolerated, and free of known drug interactions or serious adverse effects, making it a suitable candidate for long-term therapeutic strategies [18]. Some clinical studies employing its precursor β-ala have instead shown some unwanted effects consisting in paresthesia [19] or enhanced pain perception [20]. Given the broad cellular functions, spanning multiple physiological cellular processes, further strengthens the view of carnosine as a versatile and possible therapeutic target for a wide spectrum of chronic pathological conditions [21],[22], including neurodegenerative disorders [4]. Moreover, while *in vitro* studies provide crucial insights into the comprehension of the carnosine cellular and molecular mechanisms of action, animal preclinical models are essential to get the complexity of a living organism. In fact, *in vivo* systems enable a more complete evaluation of the efficacy, toxicity, and pharmacokinetics of the dipeptide, all aspects that cannot be fully addressed in a cell-based study. Furthermore, the more sophisticated genetic manipulation technique has enabled the development of animal models that closely resemble human pathological conditions, enabling the translational value of preclinical findings. Carnosine is a promising dipeptide, but its translation into clinical practice remains challenging due to significant “internal” heterogeneity related to the *in vivo* studies along with inconsistencies between the latter and clinical studies, the most important one regarding the use or not of anserine together with carnosine. These internal gaps are largely ascribable to the different types of experimental model used, the dosage administered, and the route of administration selected. In this review, we aim to (i) provide a comprehensive overview of the preclinical results obtained with carnosine administration on *in vivo* models; (ii) summarize the experimental models, dosages, protocols, and routes of administration employed; and (iii) analyze the effect of carnosine on brain with particular attention on neurodegenerative disorders. It is worth mentioning that all the studies described below were conducted using L-carnosine unless otherwise specified. Interestingly, though carnosine has consistently shown efficacy in reducing oxidative stress, neuroinflammation, and protein aggregation in neuropathological condition, there are critical knowledge gaps to be filled. Further investigation is needed to better understand carnosine effect and impact on cellular and molecular mechanisms that are central to neurodegenerative processes, such as synaptic plasticity, neuronal remodeling, and synaptic weakening, which underline neuronal dysfunction in central nervous system (CNS) disorders. Overall, the review emphasizes the urgent need for more standardized and translationally leaning studies to clarify carnosine's true therapeutic potential in the context of human diseases, with a closer eye on neurodegenerative disorders. Finally, in the discussion, we explore future perspectives and the challenges that must be addressed to effectively translate preclinical success into clinical practice.

## 2. *In vivo* administration routes and animal models

*In vivo* carnosine studies encompass a wide range of animal models, dosing treatments, and delivery routes across pathological conditions. The studies included in Table 1 were identified through searches on PubMed and Google Scholar databases, covering the period from 2004 to 2025, using the “carnosine” in combination with one or more keywords, including “animal models”, “*in vivo*”, “systemic disorder”, and “neurodegenerative disorders.” The resulting studies, after a validity check, have been subsequently categorized according to year of publication, pathology, animal model, carnosine dose, administration route, and duration of the treatment. Table 1 and the discussion of the contents are structured to first address the effects of carnosine in non-neurological conditions, such as diabetes and pulmonary dysfunction, followed by a more in-depth analysis of neurological pathologies.

**Table 1.** *In vivo* carnosine studies: Treatments, doses, and delivery routes across pathological conditions.

**Non-neurological pathologies**						
**Article**	**Pathology**	**Animal model**	**Carnosine dose** (unless otherwise indicated)	**Administration route**	**Duration**	**Note**
Lee 2005 [23]	Diabetes	Three- to four-week-old male Balb/cA mice	0.5, 1 g/L	Oral (daily) in the drinking water	4 weeks	To induce diabetes, the mice were treated with streptozotocin (STZ) (40 mg/kg BW in 0.1 M citrate buffer, pH 4.5) i.p. for 5 consecutive days.
Tanida 2005 [24]	Renal sympathetic nerve activity (RSNA) in urethane-anesthetized rats	Male Sprague-Dawley (SD) rats, weighing ∼350 g	0.01, 0.1, 1, 10, and 100 µg/0.1 ml saline	Intravenous (i.v.) injection	120 minutes	
Kurata 2006 [25]	RSNA and Ischemia/Reperfusion (I/R)-Induced Renal Injury	Male SD rats (280–320 g; 10 weeks of age)	Carnosine (1.5 and 15 nmol/rat i.v. or 1.5 and 5 pmol/rat i.c.v.) or L-histidine (5 pmol/rat i.c.v.) or N-α-Acetyl-L-carnosine [N-acetyl-β-alanyl-L-histidine] (5 pmol/rat i.c.v)	i.c.v.	45 minutes (RSNA); 24 hours (I/R-Induced Renal Injury)	Drugs were administered to ischemic-induced Acute Renal Failure (ARF) rats 5 minutes before the start of ischemia. A selective histamine H3 receptor antagonist, thioperamide (30 nmol/rat i.c.v.), which was given 10 minutes before the ischemia, eliminated the preventing effects by L-carnosine (15 nmol/rat i.v.) on ischemic ARF. In contrast, a selective H3 receptor agonist, R-methylhistamine (5 pmol/rat i.c.v.), prevented the I/R-induced Renal Injury as well as L-carnosine (5 pmol/rat) did.
Cuzzocrea 2006 [26]	Bleomycin-induced lung injury	Male CD mice (25–35 g; Harlan Nossan)	150 mg/Kg	Oral (daily)	7 days	Carnosine administered 30 minutes after the bleomycin.
Mahmoud 2006 [27]	Hypercholesterolemic	Male newzeland white rabbits (2.5–3 kg)	50 mg/Kg	Oral (daily)	6 weeks	Comparative study between carnosine and fluvastatin.
Sauerhöfer 2007 [28]	Diabetes	Cg-m+/+Lepr db/J mice (line 000642; The Jackson Laboratory, ME). Model 1: Nondiabetic nontransgenic (*db/wt*) mice. Model 2: Diabetic nontransgenic (*db/db* water) mice. Model 3: Diabetic transgenic *db/db* (*db/db* CN1+) mice. Model 4: hCN1 transgenic *db/wt* mice.	4 mmol/l L-carnosine	Oral in the drinking water (three times a week)	23 weeks (L-carnosine supplemented *db/db* mice gained significantly more weight throughout the observation period until 22 weeks of age compared with diabetic controls (*db/db* water)	In the experimental group of nontransgenic *db/db* mice was given drinking water supplemented with L-carnosine or water only (*db/db* water). L-carnosine-supplemented drinking water was replaced three times a week. Direct measurements of L-carnosine within the drinking bottles verified that l-carnosine was stable over a period of at least 5 days at room temperature (data not shown). Daily drinking volumes were determined.
Fouad 2007 [29]	IR liver injury	Male SD rats, weighing 180–200 g	250 mg/kg	i.p.	7–14 days	Pre-treatment with a single i.p. dose of carnosine, 30 minutes prior to the ischemic episode.
Kamei 2008 [30]	Diabetes	Male ICR 4-week-old mice (about 20 g)	L-carnosine (0.5 mmol/kg) Zinc-L-carnosine (0.25–1 mmol/kg)	Oral (daily)	8 weeks	Carnosine administered 1 day after the injection of STZ (200 mg/kg, i.v.), used to induce diabetes in animals.
Liu 2008 [31]	Ethanol-induced chronic liver injury	Male Balb/cA mice, 3–4 weeks old (BW of 22.1 ± 0.5 g)	0.5, 1, 2 g/L	Oral in the drinking water	3 weeks	
Mehmetçik 2008 [32]	Hepatotoxicity (thioacetamide-induced liver injury)	Female Wistar rats	250 mg/kg	i.p.	24 hours	Carnosine was co-administered with thioacetamide (TAA, 500 mg/kg) or alone. Carnosine and TAA were dissolved in 0.9 NaCl, and all intraperitoneal injections were applied to rats in a total volume of 1 mL.
Baykara 2009 [33]	I/R injury in the liver	Female Wistar albino rats, weighing 200–250 g	250 mg/kg	i.p.	4/5 hours	Carnosine and melatonin (10 mg/kg) were administered i.p. 30 minutes before ischemia and immediately after the reperfusion. The liver was reperfused for 4 hours in the groups.
Yan 2009 [34]	Hepatotoxicity: acetaminophen-induced liver injury	Five- to 6-wk-old male Balb/cA mice	0.5, 1, or 2 g/L	Oral in the drinking water	4 weeks	After 4 weeks of treatment, mice were divided into 2 groups. One group was sacrificed, and the other group was treated by acetaminophen (APAP) intraperitoneally (i.p. 350 mg/kg body weight), and killed with carbon dioxide after 24 h.
Fouad 2009 [35]	Cadmium-induced acute hepatotoxicity	Male albino mice (25–30 g)	10 mg/kg	i.p. (daily)	4 days	Carnosine administered 3 consecutive days, starting 1 day before cadmium administration.
Kamal 2009 [36]	Physiological process: Effect of PEPT2 on the disposition of endogenous and exogenous carnosine	Gender- and weight-matched wild-type (Pept2+/+) and null (Pept2-/-) mice (99% C57BL/6 genetic background) 8 to 10 wk	1 nmol/g	i.v. bolus dose	Minutes	Pharmacokinetic studies.
Renner 2010 [37]	Human cancer therapy	Female nude mice Crl: CD® -1-Foxn1^nu^	500 µl carnosine (1 M) dissolved in NaCL-solution (0.7%), ph 7.4	i.p. (daily)	24 days	All injections were performed for 6 days with a 1-day break, starting again for 6 days.
Turkcu 2010 [38]	Ethanol-induced oxidative stress	Six-month-old male Wistar rats (250–300 g)	1 mg/kg	Oral (daily)	13 days	Carnosine was administered alone or as add-on to ethanol (EtOH). EtOH + CAR group: Carnosine was administered orally 10 minutes after the ethanol was injected i.p. at a dose of 2 g/kg/day. Carnosine was dissolved in phosphate-buffered saline solution.
Aydin 2010 [39]	Treatment on prooxidant-antioxidant balance	Young (5 months) and aged (22 months) male Wistar rats	250 mg/kg	i.p. (daily)	1 month	
Aydin 2010 [40]	Thioacetamide (TAA)-induced liver cirrhosis	Female Wistar rats (BW 160–200 g)	2 g/L	Oral (daily) in the drinking water	3 months	TAA plus carnosine group received 200 mg TAA/kg BW i.p. twice a week plus 2 g carnosine/L for 3 months.
Artun 2010 [41]	Ethanol-induced oxidative stress and hepatotoxicity	Female Wistar rats	2 g/L in drinking water	Oral (daily)	4 weeks	
Riedl 2011 [42]	Diabetic nephropathy (DN)	Male Wistar rats	1g/kg BW	Oral (daily)	3 months	L-carnosine was administrated after 3 months of diabetes and treatment (in Diabetic Wistar rats).
Aldini 2011 [43]	Dyslipidaemia and renal function in Zucker obese rats	Male Zucker obese (*fa/fa*) rats (ZK) and lean littermates (*Fa/Fa*) (LN) at 5 weeks of age.	The Zucker rats received a daily dose of L-CAR or D-CAR (30 mg/kg in drinking water). For the evaluation of the pharmacokinetic profile, L-CAR or D-CAR was orally administered at a dose of 100 mg/kg to three male SD rats/test compound.	Oral (daily)	24 weeks	Approximately 3 days before treatment and while under anaesthesia, animals were fitted with a cannula implanted in the superior vena cava via the jugular vein.
Tsoi 2011 [44]	Stress-induced glucose metabolism disorder	Six-week-old male Kunming mice, weighing from 18 to 22 g	150 and 300 mg/kg	Oral/daily (carnosine was dissolved in distilled water)	7 days	
Mong 2011 [45]	Hepatic steatosis	Male 3-week-old C57BL/6 mice (16–18 g)	1 g/L	Oral (daily)	8 weeks	Water intake ml/mouse/day: Control diet 2.6 ± 1.2 (2.6 mg/d); high fat diet 5.9 ± 1.2 (6 mg/d).
Li 2012 [46]	Stress-evoked immunocompromise	Seven-week-old male Kunming and C57BL/6 mice	150 and 300 mg/kg B W	Oral (daily)	7 days	
Menini 2012 [47]	Atherosclerosis and renal disease	Adult (aged 6 weeks) female ApoE null mice (Charles River)	60 mg/kg BW in the drinking water	Oral (daily)	12 weeks	
Peters 2012 [48]	Diabetic nephropathy (DN)	Male C57BL/KsJm/Leptdb (*db/db*) mice	5 g/L in the drinking water	Oral (the estimated daily intake was 20 mg carnosine per day)	4 weeks	Treatment of *db/db* mice started at 8 weeks of age, before they had developed hyperglycemia and proteinuria.
Ansurudeen 2012 [49]	Diabetic wound healing	C57BL/KsJm/Leptdb (*db/db*) mice	100 mg/kg B W	i.p. (daily)	12 days	100 µL of carnosine (25 mg/mL in 60 % polyethylene glycol) was administered every day and was topically applied through the dressing every alternate day.
Barski 2013 [50]	Arteriosclerosis	Female ApoE-/- mice (19–23 g)	Octyl-D-carnosine 60 mg/kg/day	Oral (daily)	6 weeks	Water consumption of 2.5 mL/mouse/day.
Everaert 2013 [51]	Muscle contractility and fatigue	Male Naval Medical Research Institute (NMRI) mice (45.9 ± 5.9 g BW)	0.1%, 0.5%, or 1.8%	Oral in the drinking water	8–12 weeks	
Sahin 2013 [52]	Septic shock	SD rats	250 mg/kg	i.p.	24 hours	Rats in the treatment group received a single I.P. of carnosine 60 minutes after cecal ligation-perforation.
Kalaz 2014 [53]	Oxidative stress and tissue damage induced by D-galactose in rat liver	Male Wistar rats (200–220 g)	250 mg/kg/daily; 5 days/week	i.p.	2 months	Rats received GAL (300 mg/kg) alone or together with CAR or TAU (2.5 % w/w; in rat chow)
Giriş 2014 [54]	Hepatic steatosis and oxidative stress	Male SD rats	2 g/L in drinking water	Oral (daily)	8 weeks	
Macarini 2014 [55]	Pathophysiological mechanisms including Impairment of electron transfer chain	Twenty-four male Wistar rats (250–300 g; age 30 days)	100 mg/kg of BW	i.p.	24 hours (in acute administration) or 5 days (in chronic administration)	Male Wistar rats were divided into acute and chronic treatment groups: In the first administration, the animals received a single dose of carnosine; in the second administration, the animals received a daily dose of the dipeptide administered for five days, and 1 hour after the last injection the rats were euthanized by decapitation.
Brown 2014 [56]	Atherosclerosis	Apo E-/- mice (50% C57BL/6, 50% 129SvJ) 5–9 weeks, 24–30 g	2 g/L	Oral (daily) (water available *ad libitum*)	20 weeks	Previous studies have revealed increases in plasma carnosine at 1.0 but not 0.5 g carnosine/L drinking water over 4 weeks.
Evran 2014 [57]	Isoproterenol (ISO)-induced myocardial infarction	SD male rats (300–400 g)	250 mg/kg/day	i.p.	2–12 days	Carnosine administered 30 minutes or 10 days prior to injection with ISO. The effects of carnosine treatment on these parameters were also investigated 24 hours after the last ISO injection.
Peters 2014 [58]	Diabetes	SD rats (350 to 420 g)	1 g/kg BW	Oral (daily)	24 weeks	
Bao 2015 [59]	N/A	Pigs (Landrace × Yorkshire) with an initial BW of 57.93 ± 3.14 kg	0.1% supplemental level in basal diet	Oral (diet, *ad libitum*)	~2 months (the experimental period is not stated, but it can be approximately estimated based on the results [total weight gain divided by daily weight gain]).	Comparative and combination study with alpha-lipoic acid (0.03%). L-carnosine supplementation induced weight gain, increased serum triiodothyronine, thyroxine levels, and decreased total cholesterol and triglycerides levels.
Stegen 2015 [60]	High-fat diet-induced metabolic stress	Old male Wistar rats	1.8% carnosine in their drinking water (80 mmol/L) 1697 ± 175 mg/kg/day	Oral (daily) (water available *ad libitum*)	8 weeks	1.8% was chosen because Everaert et al. (2013) have shown that it results in a significant increase in carnosine (+57%) in extensor digitorum longus muscle in mice.
Forsberg 2015 [61]	Diabetes (glucose homeostasis)	*Db/db* mice (as a model of type 2 diabetes) and heterozygous non-diabetic mice, littermate as the control	5 g/L	Oral in the drinking water	4 weeks	Since L-carnosine was reported to be stable in the water bottles over a period of minimum 5 days at room temperature, it was chosen to replace the water every 5 days.
Wu 2015 [62]	Physiological alterations including blood glucose, cardiovascular functions	Male Wistar rats (6 weeks of age and weighing ∼160 g)	10 mg/kg	Orally (daily)	2 weeks	Rats were fed carnosine Car (for 2 weeks) before the reversal of light-dark cycle. Then rats were also subjected to a similar 12 hours experimental jet lag and sampled at 4 hours intervals on day 3 and day 5.
Menini 2015 [63]	Diabetes-induced atherosclerosis and renal disease	Six-week-old adult female Apoe-null mice were rendered diabetic by STZ and non-diabetic Apoe-null mice as controls	60 mg/kg BW D-carnosine-octylester (DCO) dissolved in the drinking water	Oral (daily)	20 weeks	Apoe-null mice were rendered diabetic by STZ and were left untreated or were treated with DCO for 20 weeks (DCO-Extended), from week 1 to 11 (DCO-Early) or from week 9 to 19 (DCO-Late). Non-diabetic Apoe-null mice served as controls.
Alsheblak 2016 [64]	Hepatic injury	Male SD rats	250 mg/kg; daily	i.p.	6 weeks	
Ahshin-Majd 2016 [65]	STZ-induced diabetic	Male albino Wistar rats, 8–10 weeks old (185–240 g)	50 and 100 mg/kg	i.p. (dissolved in normal saline)	7 weeks	The treatment of carnosine began 1 week after induction of diabetes using STZ.
Milewski 2016 [66]	TAA-induced liver failure	Adult male SD rats, weighing 250–280 g	His or Car (100 mg/kg)	i.p.	24 hours	L-histidine or carnosine were administrated 2 hours before TAA (i.p., 300 mg/kg 3 × in 24 hours intervals) injection into rats.
Kumral 2016 [67]	Doxorubicin (DOX)-induced toxicity	SD male rats that weighed 200–220 g	250 mg/kg	i.p.	12 days	Rats were treated with carnosine or carnosine + vitamin E (equals 200 mg kg^−1^α-tocopherol; once every 3 days; intramuscularly) for 12 consecutive days.
Hasanein 2016 [68]	Lead acetate-induced hepatotoxicity	Adult male Wistar rats (8 weeks old, weighing 220–250 g)	10 mg/kg	Oral (daily)	8 weeks	
Hasanein 2015 [69]	Lead-induced oxidative stress and nephrotoxicity	Adult male Wistar rats weighing 220–250 g	10 mg/kg	Intragastrically (i.g.)	8 weeks	Animals received an aqueous solution of lead acetate (500 mg Pb/L in the drinking water) and/or carnosine.
Fouad 2017 [70]	Liver carcinogenesis	SD male rats (250–280 g)	10 mg/kg/day	i.p.	2 weeks	Carnosine was administered 5 days after trichloroacetic acid (TCA) treatment (500 mg/kg/day, p.o. for 5 days).
Sun 2017 [71]	Acute Lung Injury in Sepsis	Adult male albino Wistar strain rats (160–180 g)	25 mg/kg and 50 mg/kg	Oral	30 days	
Hasanein 2017 [14]	Ameliorating nickel-induced nephrotoxicity	Adult male Wistar rats weighing 220–250 g	10 mg/kg	i.g.	21 days	Animals received NiSO4 (20 mg/kg/day i.g.) and (or) carnosine and then were evaluated for biochemical, molecular, and histopathological alterations.
Albrecht 2017 [72]	DN	BTBR (Black and Tan, BRachyuric) *ob/ob* and *ob/ob* control mice	4 mM	Oral (daily)	18 weeks	Interestingly, carnosine-administered *ob/ob* mice showed a significantly lower daily water intake towards the end of the experimental period compared with *ob/ob* control mice.
Aydin 2017 [73]	STZ-induced diabetes	Male Wistar rats (3–4 months; 220–240 g)	250 mg/kg; five times a week	i.p.	4 weeks	Carnosine was administered after 8 weeks of high fat diet (60% of total calories from fat) and 4 weeks after of STZ treatment (40 mg/kg) for a total 12-week experimental period.
Aydin 2017 [74]	Renal Function, Oxidation and Glycation Products in diabetic rats	Male Wistar rats (3–4 months; 220–240 g)	250 mg/kg; five times a week	i.p.	4 weeks	
Sahin 2018 [75]	Septic Shock	SD rats (weight, 200–300 g)	250 mg/kg (diluted into 5 mL saline)	i.p.	24 hours	Rats (in group 3) received an i.p. of carnosine 60 minutes following cecal ligation and puncture (CLP).
Iacobini 2018 [76]	The effect of FL–926–16 (a novel bioavailable carnosinase-resistant carnosine derivative) in the DN	C57BLKS/J^Lepr^ male 6-week-old diabetic *db*/*db* and control *db*/*m* mice	FL–926–16 (30 mg·kg^−1^ body weight)	Oral (daily) in the drinking water	From weeks 6 to 20 (prevention protocol) or from weeks 20 to 34 (regression protocol)	
Deng 2018 [77]	Oxidative DNA damage in bone marrow cells as a side effect of the anti-cancer alkylating agent cyclophosphamide (CTX)	Male Kun-Ming (KM) mice (18–22 g)	100 and 200 mg/kg	i.p. (after CTX treatment)	1, 5, and 10 days	
Abplanalp 2019 [78]	The deleterious effects of particulate matter exposure	Male C57BL/6 mice at 12 weeks of age	1 mg/mL	Oral (daily) (water available *ad libitum*)	6-hour exposure a day of a 9-day exposure regimen	Carnosine was administrated 1 week before the onset of exposure.
Liu 2020 [79]	CTX-induced bone marrow suppression	Male C57BL/6J mice (6–8 weeks)	1 g/kg/day	Oral (daily) (water available *ad libitum*)	8, 12, and 16 weeks	
Zhao 2020 [80]	Myocardial I/R injury prevention	Adult male C57BL/6, wild type littermates (WT) and ATPGD1-transgenic (Tg) mice	- β-alanine (20 mg/mL) for adult male C57BL/6 mice (to increase intracellular levels of carnosine); - Carnosine (±10 mg/mL) for WT mice	Oral (daily) in the drinking water	7 days	To evaluate the effect of elevated myocardial carnosine levels, these mice were subjected to coronary ligation for 30 minutes followed by 24 hours of reperfusion.
Everaert 2020 [81]	DN	Human CNDP1 TG mice were generated in the BTBR^*Wt/Ob*^ (Black and Tan, BRachyuric)	10 mM	Oral (daily) in the drinking water	20 weeks	Two interventions, aerobic exercise training and overexpression of the human carnosinase-1 (hCN1) enzyme, were tested.
Qiu 2020 [82]	Diabetic kidney disease (DKD)	Transgenic hCN1 TG BTBR^*Wt/Ob*^ and control BTBR^*Wt/Ob*^ mice	4 mM carnosine	Oral (daily) (water available *ad libitum*)	2 weeks	
Weigand 2020 [83]	Glucose homeostasis	11- and 55-week-old Cndp1-knockout (Cndp1-KO) and C57BL/6J wildtype controls mice	1 mg/mL	Carnosine substrate solution was added to 150 µL sample supernatant to test carnosinase activity	0, 10, 20, and 40 minutes of incubation	To inhibit residual renal carnosine degrading activity in Cndp1-KO mice, this assay was repeated with an additionally 0.1 mmol/L bestatin added to the carnosine substrate solution [21].
Ommati 2020 [84]	Ifosfamide (IFO) nephrotoxicity	Male SD rats (weighing 200–250 g)	250 and 500 mg/kg	i.p.	5 days	Rats were treated with IFO (50 mg/kg, i.p) alone or in combination with carnosine.
Yaqub 2021 [85]	Prepartum (ewes)	Yankasa ewes (average weight of 23.8 ± 1.21 kg and an age of 2–3 years)	100 mg/kg	Oral (daily) (water available *ad libitum*)	3 weeks	The administration started from the last 3 weeks prepartum up to the last day of gestation (between 129 ± 2 and 150 ± 2 days).
Stefani 2021 [86]	Myocardial infarction	Male Wistar rats [weighing between 220 and 300 g (approximately 70–90 days of age)]	β-alanine and L-histidine orally (250 mg/kg per day)	Oral (daily) (via gavage in 1.0 mL of distilled water)	8 weeks	Animals were submitted to myocardial infarction. Supplementation occurred at the same time of the day in each training session.
Riger 2021 [87]	Initial stage of non-alcoholic fatty liver disease	Male Wistar rats with initial BW 150 ± 10 g within 8 weeks	High-calorie choline-deficient diet (HCCDD) with the addition of carnosine (75 mg/kg BW)	Oral (daily)	N/A	
Gonçalves 2021 [88]	Cardiac dysfunction (the impairment of contractile function)	Male Wistar CARNS1^−/−^ rats (knockout, 4 months-old) and their wild type (WT) controls	1.8% in the drinking water	Oral (daily)	12 weeks	
Schwank-Xu 2021 [89]	Hepatic Steatosis in Diabetic Conditions	12-week-old *db/db* mouse model of type 2 diabetes mellitus (BKS.Cg-Dock7m+/+LeprdbJ) and heterozygote normoglycemic C57BLKS/J littermates (C57B)	20 mM	Oral in the drinking water	10 days	
Nooh 2021 [90]	Infertility related to chemotherapeutic agents		250 mg/kg/day	i.p.	5 weeks	Group I was the control. Group II received carnosine; Group III received CHOP: CTX (27 mg/kg/cycle), DOX (1.8 mg/kg/cycle), and vincristine (0.05 mg/kg /cycle) by i.p. plus oral prednisone (1.47 mg kg-1 day-1/cycle). Group IV received carnosine plus CHOP.
Zhu 2021 [91]	DN	Male C57BL/6J mice (6–8 weeks of age) STZ-induced diabetes	1000 mg/kg	Drinking water	12 weeks	Experimental groups: control group, control + carnosine group, STZ group, and STZ + carnosine group.
Busa 2022 [92]	Knee osteoarthritis (OA)	Male Wistar rats (8 weeks old)	0.5 and 1.0 g/kg/day	Oral administration	12 weeks	
Rodriguez-Niño 2022 [93]	Diabetic kidney disease (DKD)	Wild-type, diabetic BTBR^*wt/ob*,^ 6-week old male mice	45 mg/kg	Drinking water	18 weeks	Mice were either subjected to carnosine supplementation or genetically manipulated by overexpressing hCN1
Park 2022 [94]	Idiopathic Pulmonary fibrosis (IPF)	C57BL/6 mice (8 weeks/male, 22–25 g)	150 mg/kg	Oral administration	2 weeks	The mice were i.t. injected with BLM (3 mg/kg) and L-carnosine (150 mg/kg) was orally administrated for 2 weeks.
Zharikov 2022 [95]	Urate nephrolithiasis	Male Wistar rats	15 mg/kg	i.g.	10 days	
Ommati 2023 [96]	Cholestasis-induced injury	Male SD rats (250 ± 20 g)	100 and 500 mg/kg	i.p.	28 days	Bile duct ligation (BDL) to induce cholestasis.
Zhang 2023 [97]	DN	C57BL/6 J mice, 6–8 weeks of age	1000 mg/kg	Oral in the drinking water	16 weeks	Mice were injected intraperitoneally with 50 mg/kg of STZ daily for 5 days to induce diabetes. Fer-1 (delivered i.p.) 5 mg/kg; carnosine delivered at 1 g/kg dose of drinking water.
Rathor 2023 [98]	Skeletal muscle protein loss	Male SD rats (BW: 200 ± 20 g)	50 mg/kg	Oral route using a gastric cannula once daily	3 days	Efficacy of nutritional supplementation of β-alanine (450 mg/kg) and L-carnosine (50 mg/kg) in ameliorating the hypobaric hypoxia-induced muscle protein loss.
Luo 2023 [99]	Acute kidney injury	Male C57BL/6 J mice (age, 6–8 weeks; weight, 18–22 g	2g/kg	i.p.	3 days before and 1 day after cisplatin injection	Pyroptosis is involved in cisplatin-induced acute kidney injury.
Berdaweel 2023 [100]	Obesity-related cardiomyopathy	Wild-type (WT) and GPx4^+/−^male mice	(80 mM)	Oral (daily) in the drinking water	9 weeks starting from the 7th week after beginning the diet	Wild-type (WT) and GPx4^+/−^male mice were randomly fed a standard (CNTL) or high fat high sucrose diet (HFHS) for 16 weeks.
Moreto 2023 [101]	Nonalcoholic fatty liver disease (NAFLD)	Male Wistar rats (8 weeks old)	250 mg/kg/day	i.p.	5 weeks	The administration of L-carnosine reversed liver steatosis. The protein profiles of NAFLD liver group and carnosine NAFLD liver group were evaluated by label-free proteomics approach (2531 proteins were identified).
Grandini 2024 [102]	Metabolic dysfunction-associated steatotic liver disease (MASLD)	Male Wistar rats	250 mg/kg	i.p.	4 weeks	
D'Amato 2024 [103]	Chronic obstructive pulmonary disease (COPD)	C57BL/6J mice	10, 50, or 100 mg/kg/day	Inhaled	12 days	The mice were exposed to 3R4F reference cigarettes with filters removed for approximately 60 min, twice daily, 4 hours apart, for 12 consecutive days.
Drenjančević 2024 [104]	Oxidative stress	SD rats (8–10 weeks old, both sexes)	150 mg/kg/day	Oral administration (gavage)	7 days	High-salt (HS)-intake-related increase of oxidative stress. Experimental groups: CTRL (control group, 0.4% NaCl), HS group (rats fed 4% NaCl in the rat chow for 7 days), CTRL + carnosine group (rats administered oral carnosine supplementation, 150 mg/kg/day for 7 days by oral gavage), and HS + carnosine group (rats fed HS diet and receiving oral carnosine supplementation).
Xu 2025 [105]	Focal segmental glomerulosclerosis (FSGS)	WT and TGRNeph-hAT1 transgenic rats with sex-dependent FSGS	4 nM	Oral (daily) in the drinking water	20 weeks	Carnosine supplementation ameliorated glomerular hypertrophy.
Meng 2025 [106]	N/A	3-month-old male crossbred lambs (Dorper ♂ × Small Tail Han ♀) with an average BW of 30 ± 5 kg	400 mg/kg	Oral (diet)	60 days	Lambs were randomly divided into two groups. Animals in the control group were fed only the basal diet. In the experimental L-carnosine group, L-carnosine was added to the basal diet.
Li 2025 [107]	Oxidative stress	Zebrafish larvae, chick embryos	Zebrafish larvae: 0.1, 0.5, 1.0, 1.5, and 2.0 mM. Chicken embryos: 2.5, 5 and 7.5 µmol.	Zebrafish larvae: Incubation in 48-well plates. Chicken embryos: Injection.	Zebrafish larvae: 5 days. Chicken embryos: 12 days.	

**Neurological pathologies**						
**Article**	**Pathology**	**Animal model**	**Carnosine dose** (unless otherwise indicated)	**Administration route**	**Duration**	**Note**

Tomonaga 2004 [108]	Carnosine effects: inhibited food intake and hypoactivity	Day-old male chicks (Julia strain)	3.2 and 6.4 µmol	Intracerebroventricular (i.c.v.) injection	30, 60, 120, and 240 min	Experiment 1: Chicks (5-day-old) were injected carnosine (3.2 and 6.4 mol). Experiment 2: Chicks (6-day-old) were injected carnosine (6.4mol) Experiment 3: Carnosine (0.8, 3.2 and 6.4 mol).
Tomonaga 2005 [109]	Carnosine effects: hyperactivity	5 or 6 days-old male chicks (Julia strain)	3.2 µmol	i.c.v.	5, 10, and 15 min	iNOS inhibitors were also used. Experiment 1: Chicks were injected with carnosine plus L-NAME (400 nmol). Experiment 2: Carnosine (3.2 µmol) plus L-NAME (200 or 400 nmol). Experiment 3: Carnosine (3.2 µmol) plus D-NAME (400 nmol). Experiment 4: Carnosine (3.2 µmol) plus L-NIL (400 nmol).
Zemke 2005 [110]	Mouse model of stroke	Male C57BL/6J mice	100 mg/kg and 500 mg/kg carnosine	Intraperitoneal (i.p.) injection	24 hours	Administered by intraperitoneal injection to male C57BL/6J mice 30 minutes prior to permanent occlusion of the middle cerebral artery.
Fedorova 2005 [111]	Prenatal hypoxia	Male and female 22 and 30-day-old rats	100 µg/kg body weight (BW)	Oral (daily) in the drinking water	35 days	
Dobrota 2005 [112]	Ischemic injury (after-stroke-effect)	Model 1: Wistar rats (280–300 g) Model 2: Mongolian gerbils (65–75 g)	100 mg/kg BW	i.p.	7–14 days	Model 1: Modification to the 4-vessel occlusion model (16) was made, and animals were exposed to 3-vessel occlusions (both common carotid arteries and the left arteria vertebralis) for 15 min. Model 2: Two-vessel occlusion (left and right arteria carotis communis) producing brain ischemia was followed by long-term reperfusion in this animal group.
Jin 2005 [113]	Amygdaloid-kindled seizures	Male SD rats (220–300 g)	(500, 1000, and 1500 mg/kg)	i.p.	0.5, 1, 2, and 4 hours	The protective effect of carnosine (1500 mg/kg) was completely antagonized by histamine H1-antagonists pyrilamine (2, 5 mg/kg, i.p.) and diphenhydramine (5, 10 mg/kg, i.p.) but not by histamine H2-antagonist zolantidine even at a high dose of 10 mg/kg.
Wu 2006 [114]	Pentylenetetrazole-induced kindled	Male SD rats (220–300 g)	200, 500 mg/kg	i.p.	30 min	Carnosine was injected 2 hours before PTZ treatment. Chemical kindling was elicited by repeated intraperitoneal injection of PTZ (35 mg/kg) once every 48 hours until the occurrence of Stage 4–5 seizures, and the seizure activity of kindling was recorded for 30 min.
Tsuneyoshi 2007 [115]	N/A	5- or 6-old-day male chicks (Julia strain)	2.8 µmol of carnosine	i.c.v.	10 min	Comparative study between carnosine and other β-alanyl dipeptides.
Rajanikant 2007 [116]	Neuronal damage, infarct formation, endogenous antioxidant status, matrix metalloproteinase activity.	Male C57BL/6 mice (mouse model of permanent focal cerebral ischemia)	L-carnosine was dissolved in 9g/L sterile saline (100 mg/mL). 100, 500, and 1000 mg/kg administered 30 minutes before ischemia, or 1000 mg/kg administered 2 or 4 hours after ischemia supplemented by doses of 500 mg/kg every 6 hours, or 1000 mg/kg administered 30 minutes before ischemia supplemented by doses of 500 mg/kg every 6 hours.	i.p.	24 hours	Carnosine significantly decreased infarct size and neuronal damage when administered at time points both before and after the induction of ischemia.
Zhu 2007 [117]	Pentylenetetrazol-induced seizures	HDC-KO (histidine decarboxylase-deficient) and its WT (C57BL/6 strain) male mice	200, 500 or 1000 mg/kg	i.p.	4 hours	Carnosine was injected 1 hour before PTZ injection. The effects of carnosine (500 mg/kg) were time-dependent and reached a peak at 1 h.
Tanida 2007 [118]	Effects of injection of L-carnosine on sympathetic nerve activity	Male Wister rats, (300–350 g)	0.01, 0.1, 1, 10 and 100 µg	Intralateral cerebral ventricular (LCV) injection	120 min	
Tomonaga 2008 [119]	Depression (carnosine-induced antidepressant-like activity)	Male Wistar rats (six weeks old)	1.4 mmol/kg → 316,722 mg of carnosine/kg	Oral (10 ml CBX)	240 min	
Min 2008 [120]	Permanent focal ischemia	C57BL/6 mice (22–27 g)	Carnosine 1000 mg/kg or 1000 mg/kg N-acetyl carnosine	i.p.	1 day	Administered to mice 30 minutes before induction of permanent middle cerebral artery occlusion (MCAO).
Derave 2008 [121]	Senescence	Male SAMP8/Ta (senescence-accelerated mice prone)	100 mg/kg BW	Oral in the drinking water	50 weeks (from 10 to 60 weeks of age)	Mice were investigated at 10, 25, or 60 weeks of age.
Kozan 2008 [122]	Epileptiform activity	Epilepsy model in Wistar rats	125, 250, 500, and 1000 mg/kg	i.p.	90 min	30 minutes after penicillin injection, the doses of 125, 250, 500, and 1000 mg/kg of carnosine were administered i.p., and 90 minutes before penicillin injection, a dose of 500 mg/kg carnosine were administered i.p.
Pekcetin 2009 [123]	Transient cerebral ischemia	Female Wistar rats weighing between 200–250 g	250 mg/kg	i.p.	24 hours or 1 week	Transient ischemia was induced by occlusion of right common carotid artery of rats for 30 minutes and reperfusion for 24 hours or 1 week. Carnosine or saline solution were administered 30 minutes prior to experiment.
Feng 2009 [124]	Neuronal excitation and inhibition	Adult SD rats (200–350 g)	25, 50, and 100 mmol/L	Oral	20–30 min	To identify the relatively quick effects and slow effects separately, the PS (population spikes) responses during the first 5 minutes and late 20–30 minutes periods following the application of carnosine were evaluated.
Aydin 2010 [125]	Aging process	Young (5 months) and aged (22 months) male Wistar rats	250 mg/kg	i.p. (daily)	1 month	
Shen 2010 [126]	Permanent cerebral ischemia	Wild-type (WT) and HDC-KO male mice (C57BL/6 strain) (22–30 g)	250–500–750 mg/kg [Carnosine was dissolved in 9 g/L sterile saline (100 mg/ml)]	i.p. (daily)	24 hours	Carnosine was administered 30 minutes before ischemia.
Tsai 2010 [127]	1-methyl-4-phenyl-1,2,3,6-tetrahydropyridine (MPTP)-treated mice	Three- to four-week-oldmale C57BL/6 mice (25 g)	0.5, 1, or 2 g/L in water (Water Intake 2 mL/mouse/day)	Oral (daily) (water available *ad libitum*)	4 weeks	After 4 weeks of care with carnosine, mice were treated by daily subcutaneous injection of vehicle saline or MPTP (24mg/kg) for 6 consecutive days.
Zhang 2011 [128]	Hypoxia-ischemia	SD rat pups of either sex that weighed between 12 and 18 g	250 mg/kg	i.p.	24 hours	Hypoxia-ischemia was induced in rats on postnatal day 7 (P7). Carnosine was administered 30 minutes prior to hypoxia-ischemia induction.
Di Paola 2011 [129]	Acute spinal cord injury (SCI)	Male Adult CD1 mice (25–30 g)	150 mg/kg D-carnosine/L-carnosine	i.p.	24 hours	L-carnosine and D-carnosine were administrated 1 hour and 6 hours after SCI.
Corona 2011 [130]	Alzheimer's disease	One month old male 3xTg-AD mice	10 mM	Oral (daily)	11–13 months	
Faddah 2012 [131]	Brain damage [traumatic brain injury (TBI)]	Male Wistar albino rats weighing 40 to 60 g (forty days old)	200 mg/kg	i.p. (daily)	7 days	Carnosine was administered for 7 consecutive days following TBI.
Ma 2012 [132]	Subcortical ischemic vascular dementia	Eight-week-old wild-type (WT, C57BL/6 strain) and HDC-KO male mice weighing 22–30 g	200 mg/kg	i.p. (carnosine was dissolved in sterile saline)	32 days	C57BL/6 mice were subjected to permanent occlusion of the right unilateral common carotid arteries (rUCCAO) and treated with carnosine or histidine (200 or 500 mg/kg) that were administered 30 minutes before surgery and every other day until the mice were sacrificed.
Ma 2012 [133]	Subcortical ischemic vascular dementia	Eight-week-old wild-type (WT, C57BL/6 strain) and HDC-KO male mice weighing 22–30 g	100, 200 or 500 mg/kg	i.p.	37 days	Adult male WT mice received rUCCAO and were administered with saline, carnosine, or histidine (200 or 500 mg/kg), 30 minutes before surgery and every other day until the mice were sacrificed.
Çoban 2013 [134]	Oxidative stress in some tissues of aged rats	Young (5 months) and aged (22 months) male Wistar rats	250 mg/kg	i.p.; 5 days per week	2 months	It investigated the effects of CAR + vitE and betaine treatments on oxidative and antioxidative status in liver, heart and brain tissues of aged rats.
Herculano 2013 [135]	Alzheimer's disease	B6C3-Tg (AβPP swe/PSEN1dE9) 85Dbo/J AD model animals. [These animals express the Swedish variation of the phenotype, presenting both a chimeric human AβPP transgene (Mo/HuApp695swe) and human PS1 transgene (missing exon 9)].	5 mg/kg	Oral in the drinking water (daily)	6 weeks	The treatment with carnosine was given a steady dosage of carnosine diluted in de-ionized autoclaved drinking water at the concentration of 1 g/L. This treatment began 2 weeks after the initial feeding with HFD and kept until the end of the experiments.
Wang 2013 [136]	Acute focal cerebral ischemia	SD rats (250–300g)	500, 750 and 1000 mg/kg	i.p. (30 minutes before operation and very 6h thereafter)	12, 24 and 72 hours	The study included 2 stages: 1) Multiple doses of L-carnosine and 2) a single dose of L-carnosine.
Bae 2013 [137]	Ischemic brain damage	Adult male SD rats weighing 250 to 300 g	100, 500, 1000, or 2000 mg/kg B W	i.v. bolus injection	24 hours	Saline, carnosine, or histidine (1000 mg/kg B W) were administered over 3 minutes into the lateral tail vein at 30 minutes prior to the occlusion of the middle cerebral artery. Blood samples were drawn from a femoral vein catheter before administration and at 15 min, 1 h, 3 h, 6 h, 12 h, and 24 h post-administration of carnosine.
Bae 2013 [138]	Ischemic stroke	Adult male SD rats	(500 to 2000 mg/kg)	i.v.	14 days	
Park 2014 [139]	Early stage of rodent stroke model	Rats. N.I.	100, 250, and 500 mg/kg	i.p.	2 hours	Rats with vehicle and carnosine treated groups were administered by i.p. injection 30 minutes before surgery.
Zhang 2014 [140]	Hypoxia-ischemia	Postnatal day 7 SD rats	250mg/kg	i.p.	72 hours	Hypoxia-ischemia was induced in rats on postnatal days 7–9 (P7–9). Carnosine was administered at 0 h, 24 h, and 48 h after hypoxia-ischemia was induced.
Ji 2014 [141]	I/R injury	C57BL/6 J mice, 3 months of age (20–25 g in weight)	1000 mg/kg	i.p.	2 weeks	L-Carnosine was administered to mice 30 minutes before induction of retinal ischemia. Retinal ischemia was induced by constant elevation of intraocular pressure (100–110 mmHg) for 60 minutes in C57BL/6 J mice pretreated with carnosine or saline.
Inozemtsev 2014 [142]	Effects of carnosine on learning and memory of animals with negative reinforcement with an electric pain stress.	White rats weighing 250–300 g	100 mg/kg	i.p.	8 days	The animals of the experimental group were i.p. injected with carnosine 1 hour prior to each experiment.
Albayrak 2015 [143]	Acute SCI	SD rats (280–300 g)	150 mg/kg at the first hour and then at 6 hours dose regimen	i.p. (150 mg/kg at the first hour and then at 6 hours dose regimen)	24 hours	
Dai 2015 [144]	Febrile seizures	WT and HDC-KO C57BL/6J mice pups	100, 200, 500 mg/kg	i.p. (1 hour before hyperthermia)	Hours (not specified)	
Macedo 2015 [145]	Various aspects of brain bioenergetics (respiratory chain complexes and citric acid cycle enzyme activities)	Male Wistar rats (80–100 g)	100 mg/kg	i.p. (single dose)	24 hours	
Russo 2015 [146]	Retinal ischemic injury	Adult male Wistar rats (280–330 g)	0.036 µmol/eye. (L-carnosine was prepared in sterile water at 100 mM concentrations)	Intravitreal injection	1 hour of reperfusion/7 days following the insult.	L-carnosine administration, alone or in combination with homotaurine (0.059 µmol/eye), was performed 1 h before and following the 50 minutes of ischemia. The duration of the injection (3 µL/eye) was 3 minutes in all instances.
Zhang 2015 [147]	Subarachnoid hemorrhage (SAH)-induced early brain injury (EBI)	Male SD rats (weighing 280–350 g)	(0.1 mL, 1000 mg/kg)	i.p. (daily)	48 hours	
Afshin-Majd 2015 [148]	Parkinson's disease	Adult male Wistar rats, weighing 230–280 g	250 mg/kg twice at an interval of 24 h	i.p. (twice at an interval of 24 h)	1 week	Carnosine was administered two times a day before the surgery, with the last injection being 1 h presurgery.
Aydın 2016 [149]	D-galactose (GAL)-treated model	Male Wistar rats (200–220 g)	250 mg/kg/daily; 5 days per week	i.p	2 months	Rats received GAL (300 mg/kg) alone or together with carnosine or taurine (2.5 % w/w; in rat chow).
Ma 2016 [150]	White matter damage caused by subcortical ischemic injury	Male C57BL/6 mice (specific-pathogen-free/viral-antibody-free), 8-weeks-old, weighing 22–30 g	200, 500 or 750 mg/kg	i.p.	37 days	The animals were treated with carnosine by i.p. 30 minutes before injury and every other day after injury. However, carnosine at the higher dose of 750 mg/kg did not have the same effects as the 200 and 500 mg/kg doses.
El-Baky 2016 [151]	Closed Head Injury (CHI)	30-day-old male Wistar albino rats weighing 50–70 g	200 mg/kg	i.p.	7 days	Carnosine was administered immediately after truma, and for a period of 7 days following CHI.
Banerjee and Poddar 2016 [152]	Aging	Male albino Wistar strain rats	2.0 µg/kg/day	Intrathecally (i.t.)	21 days	
Al-Rasheed 2016 [153]	Hypoxic Rat Model	Wistar adult male albino rats weighing 170–200 g	250 mg/kg BW	i.p. as a single dose	25 hours	Carnosine was administered 24 hours before NaNO_2_ injection, and after one hour, the rats were sacrificed.
Kaneko 2017 [154]	Alzheimer disease	B6C3-Tg (APPswe/PSEN1dE9) 85Dbo/J AD-model mice	Anserine at 2.0 g/L (equivalent to 10 mg/mouse)	Oral (daily) (water available *ad libitum*)	8 weeks	
Zhao 2017 [155]	Salsolinol-induced Parkinson's disease	Male albino rats (weighing 180–200 g)	50 or 100 µg/mL	Oral	72 hours	Administration of 50 µg salsolinol + 50 µg carnosine or 100 µg salsolinol + 100 µg carnosine.
Keskin 2017 [156]	I/R injury	SD rats (250–300 g)	250 mg/kg	i.p. (10 minutes before completion of the ischemia period)	Minutes	
Stvolinsky 2017 [157]	Focal cerebral ischemia-reperfusion	Wistar rats	150 mg/kg of body mass	Oral administration (daily)	7 days	A focal ischemia in Wistar rats induced by the 60 min-occlusion of the middle cerebral artery with the following 24 h-reperfusion was used. Animals received carnosine mixed with ration in a daily dose for 7 days before surgery.
Xie 2017 [158]	Intracerebral hemorrhage (Oxidative Stress and Apoptosis)	Male SD rats (weighing 270–300 g, 12 weeks old)	1000 mg/kg	i.p.	72 hours	
Aydin 2018 [159]	Oxidative stress and Advanced Glycation End products (AGE) in GAL-induced aging	Male Wistar rats (200–220 g)	250 mg/kg/daily, 5 days per week	i.p	2 months	
Devyatov 2018 [160]	Focal cerebral I/R	Wistar rats	150 mg/kg	Oral administration (daily)	7 days	Animals received carnosine for 7 days before the temporary occlusion of the middle cerebral artery (MCA), performed for 60 min. At 24 hours after the onset of ischemia, the effect of carnosine on the area of the necrotic core was evaluated in animals.
Fedorova 2018 [161]	Focal Ischemia	Male Wistar rats (age 12–14 weeks)	50 and 500 mg/kg	i.p.	24 hours	Carnosine was administered intraperitoneally according to the following scheme: 15 minutes after surgery animals received half of the experimental dose of carnosine, the other half was administered 2 hours and 15 minutes after surgery.
Wang 2018 [162]	Cerebral ischemia injury	Male SD rats (age, 10–12 weeks; weight, 280–320 g)	180 mg (in rats with BW of 300 g)	Oral gavage (daily)	7 days	The researchers aimed to determine the mechanism of the protective effect of beef decoction (BD) with carnosine against it. The carnosine content in BD was 36 mg/ml.
Barca 2018 [163]	Diabetic (pancreas and brain of STZ-Treated Mice)	Inbred male C57BL/6JB6 mice (12 weeks old)	1 g/L (in sterile deionized H2O)	Oral (daily) in the drinking water	2 weeks	Carnosine detection in the pancreas and brains of mice that underwent STZ treatments.
Qi 2018 [164]	Brain oxidative damage in a pentylenetetrazole-induced epilepsy model	Female albino rats weighing 220–240 g	L-Homocarnosine (1mM); L-Carnosine (1mM)	Orally	45 days	This study investigated the protective effect of L-homocarnosine, L-carnosine, and anserine (HCA) on seizure-induced brain injuries.
Tiwari 2018 [165]	Vascular dementia	Male and Female Wistar rats weighing 200–300 g	200 and 400 mg/kg	i.p. (daily)	From 6^th^ to 9^th^ day	Donepezil and Carnosine were administered i.p. in sterile saline.
Colín-Barenque 2018 [166]	Impairment of olfactory function (such as Parkinson's and Alzheimer's diseases)	CD-1 male mice weighing 35 ± 2 g (2 months of age)	1 mg/kg	Oral (daily)	4 weeks	Carnosine treatment: Vanadium pentoxide (V_2_O_5_) inhalation plus orally administered carnosine simultaneously and orally administered carnosine.
Bermúdez 2018 [167]	Parkinson's disease (oxidative stress and mitochondrial dysfunction)	Two months old WT and Thy1-aSyn mice	• 2 mg/day for intranasal (IN) • 50 mg/day based on water consumption	IN or oral (daily)	2 months	Carnosine was applied in 10 µL of sterile dd H_2_O).
Bermúdez 2019 [168]	Parkinson's Disease	Thy1-aSyn (TG) mice	2 mg/day (2 mg in 10 µL, which is near the limit of solubility and is without adverse effects)	IN	2 months	L-carnosine (>98%) was obtained from Acros Organics via Thermo Fisher (Morris, NJ, USA).
Jain 2020 [169]	Ischemic strokes	6 to 8-week old male C57bl/6J (20–25 g)	L- and D-carnosine (100, 500 or 1000 mg/kg)	i.p.	48 hours	To determine the relative cerebroprotective potential of D- and L-carnosine in transient focal ischemic damage, MCAO was induced for 60 min. The efficacy of both L- and D-carnosine were also tested when administered intravenously 2 hours post-transient-MCAO.
Dai 2020 [170]	Aging	Three-month-old male senescence-accelerated mouse prone 8 (SAMP8) mice	Carnosine 100 or 200 mg/kg/day	i.g.	6 weeks	
Ommati 2020 [171]	Mn-induced neurotoxicity	Male C57BL/6 mice	10, 50 and 100 mg/kg	i.p.	8 days	Mice received Mn (100 mg/kg, s.c) alone and/or in combination with carnosine.
Virdi 2020 [172]	Cerebral ischemia (ischemic postconditioning)	Albino mice of either sex (20–25 g)	500 mg/kg	i.p. (carnosine was dissolved in distilled water)	24 hours	
Devyatov 2020 [173]	Focal Ischemia	Wistar rats	150 mg/kg	Oral administration (daily)	7 days	The animals received hesperetin (50 mg/kg) and carnosine included in the diet of daily doses for 7 days before ischemia induction.
Attia 2020 [174]	Hemic hypoxia	Fifty Wistar adult male albino rats weighing 170–200 g	250 mg/kg	i.p.	26 hours	Hypoxic rats were pre-treated with carnosine, or L-arginine (200 mg/kg i.p.), or both. Carnosine and L-arginine were administered 24 hours and 1 hour before sodium nitrite injection.
Kim 2021 [175]	Ischemic strokes	Adult male SD rats	1000 mg/kg	i.v. to the tail	24 hours	Carnosine was administered 3 hours after ischemia induction.
Brown 2021 [176]	Parkinson's disease (motor dysfunction and impaired olfaction)	Thy1-aSyn (a model of PD) and wild-type mice	(0.0, 2.0, or 4.0 mg/day)	IN	8 weeks	
Banerjee 2021 [177]	Aging-induced proteinopathies	18- and 24-months male albino Wistar rats	2.0 µg/kg/day	i.t. injection	21 days	
Łochyński 2022 [178]	Neuromuscular diseases associated with aging	Male Wistar rats aged 15 months	1000mg/L (~ 46.0 mg/kg BW/day)	Drinking water	10 weeks or 34 weeks	Control plus two experimental groups in which 0.1% carnosine supplementation was performed for the last 10 weeks or for 34 weeks.
Arslan 2022 [179]	Exposure to a 900 Mhz electromagnetic field (EMF).	16-week-old female Wistar Albino rats weighing 200–250 g	Carnosine administered at low (10 mg/kg/day) and high (100 mg/kg/day) doses	i.p.	28 days	Control: Not exposed to any material, EMF, or carnosine injection. EMF group (EMGG): Exposed to EMF (900 MHz) 1 hour daily over 28 days. EMFG plus carnosine (CG): Exposed to EMF (900 MHz) 1 hour daily over 28 days, carnosine administered at low (10 mg/kg/day) and high (100 mg/kg/day) doses with i.p. injection provided 30 minutes before exposure.
Peng 2022 [180]	Diabetic encephalopathy (DE)	*db/db* mice	100 mg/kg	i.p./saline oral	8 weeks	
Tsuji 2022 [181]	Autism spectrum disorder (ASD)	C57BL6/N wild-type and CD157KO mice	0.09 g/100 mL	Drinking water	10 weeks	
Hegazy 2022 [182]	Glucose dismetabolism Sporadic Alzheimer's disease (sAD)	Adult male Wistar rats (200–220 g)	100 mg/kg/day	Oral gavage	5 weeks	5 µL of either vehicle (0.9% saline for the control group) or STZ (3 mg/kg) dissolved in the same vehicle was slowly injected into the left ventricle.
Ndolo 2023 [183]	Diabetes Neurodegenerative diseases	SD rats	low (100 mg/kg), medium (300 mg/kg), and high (900 mg/kg)	Intra-gastric	12 weeks	High-fat diet (HFD) and one intraperitoneal injection of 30 mg/kg STZ, three intragastric carnosine treatment.
Hu 2023 [184]	Brain Stroke	mice	1000 mg/kg/day		Daily pre-treatment for 2 weeks and then 1 and 5 days after reperfusion	Transient MCAO mouse model for 60 minutes and continuously treated with saline or carnosine for additional 1 and 5 days after reperfusion.
Rivi 2024 [185]	Inflammation	Pond snails *Lymnaea stagnalis* (six- month-old with shell lengths of 20–25 mm)	100 µM, 1 mM, or 10 mM	Carnosine dissolved in artificial pond water	1 hour	Exposure to 1 mM carnosine before training enhanced memory formation and neuroplasticity. Moreover, pre-exposure to 1 mM carnosine before LPS administration (approximately 8 mg/kg, injection) reversed the memory deficit brought about by inflammation.
Shen 2024 [186]	Postoperative cognitive dysfunction (POCD)	24-month-old male SD rats	250 mg/kg	i.p.	Half an hour before surgery	POCD model by exploratory laparotomy in 24-month-old male rats.
Chern 2025 [187]	Alzheimer's disease	Wild-type zebrafish (*D. rerio*)	10 mM	Tank with 10 mM CAR	30 days, 4 hours each day	4 experimental groups: 1. Fish exposed to 100 nM OKA, for 9 consecutive days to induce AD-like pathology. 2. Fish administered with 10 mM CAR for 30 consecutive days prior to a nine-day exposure to 100 nM OKA, 3. Fish administered with 10 mM CAR only for 39 days, 4. Healthy fish with no treatment.

During the last two decades, almost 200 *in vivo* studies have been published describing the therapeutic potential of carnosine. In neurological disease models, intranasal or intracerebral routes have been used as they provide direct access to the CNS. For example, intranasal carnosine (approximately 2–4 mg per day for 8 weeks) significantly improved motor function and reduced α-synuclein aggregation in a transgenic mouse model of Parkinson's disease (PD) (Thy1-aSyn), highlighting effective brain delivery and preventing the peripheral degradation due to circulating carnosinases [176]. By contrast, systemic carnosine injections in toxin-based Parkinsonian rodent models (e.g., 6-OHDA-lesioned rats or MPTP-treated mice) have been shown to attenuate dopaminergic neuron loss, elevate antioxidant enzyme levels, and dampen neuroinflammation in the striatum [148]. In Alzheimer's disease (AD) models, carnosine has been given orally, yielding reductions of amyloid-β (Aβ) accumulation and expression of neuroinflammatory markers [182], and a reduction of oxidative damage with improved cognition. Parenteral and intravenous carnosine treatments have demonstrated promising results in experimental stroke models when administered within the critical therapeutic window following vessel occlusion. It reduced infarct size and enhanced neurological recovery outcomes [169],[175]. Beyond neurodegenerative models, carnosine has demonstrated therapeutic efficacy also in murine models of seizure and autism, further underscoring its broad preclinical therapeutic scope [163],[180]. Carnosine's protective effects extend beyond the brain, as demonstrated by numerous researchers investigating its role in metabolic diseases and inflammatory conditions. In chronic metabolic and cardiovascular models, carnosine has been frequently delivered orally through drinking water or diet supplementation, with diabetic mice typically receiving high-dose treatments (often hundreds of mg per kg body weight per day). For instance, carnosine added to drinking water (~1 g/kg/day) over several months alleviated diabetic nephropathy, as evidenced by reduced albuminuria, retained podocyte integrity, and suppression of pro-fibrotic signaling in the kidneys [79]. Carnosine has also shown anti-inflammatory and antioxidant properties, exerting positive effects in various pathophysiological conditions, including autoimmune diseases [174]. In rodent models of acute lung injury (lipopolysaccharide-induced) and chemical nephrotoxicity, carnosine treatment demonstrates also immunomodulatory capacity through significant reductions in tissue tumor necrosis factor α (TNF-α), interleukin-16 (IL-6), and oxidative stress levels [71].

## 3. Non-neurological pathologies: Cancer, cardiovascular disease, diabetes, and metabolic syndrome

A growing body of literature evidence suggests that supplementation with carnosine, or its rate-limiting precursor β-alanine, can ameliorate different aspects of metabolic dysregulation that occur in diabetes and its related conditions as well as in other pathologies such as cardiovascular disease, cancer, lungs, or kidney dysfunction. L-carnosine acts by targeting key pathogenic pathways, including mitochondrial dysfunction, oxidative stress, inflammation, and impaired metabolic regulation [23].

Carnosine has been shown to enhance hepatic [Coenzyme Q (CoQ) gene expression and biosynthesis in a model of type 2 diabetes (*db/db* mice)]. Indeed, the administration of L-carnosine with CoQ improves mitochondrial function, reducing reactive oxygen species (ROS) production and mitigating cellular oxidative stress [89]. In the same model of type 2 diabetes (*db/db* mice), Peng *et al*. demonstrated that oral administration of carnosine (100 mg/kg for 8 weeks) also ameliorated cognitive impairment by reducing neuronal oxidative stress and inflammation, including inducible nitric oxide (iNOS) expression and by modulating the Sirtuin 6/endoplasmic reticulum (ER) stress pathway in the hippocampus [180]. Consistent with the ability of carnosine to ameliorate cognitive impairment, it is worth mentioning the work on the activation of glutamatergic excitatory mechanisms and the related enhancement of the cognitive potential of the neural network promoted *in vitro* by carnosine [188].

Similarly, in streptozotocin (STZ)-diabetic rats, as shown by Ahshin-Majd *et al*. [65], chronic treatment with carnosine improved cognitive performance. In this work, the authors further explored the molecular mechanisms underlying carnosine's neuroprotective effects, showing a reduction of nuclear factor-kappaB (NF-κB), TNF-α, and glial fibrillary acidic protein (GFAP) in the hippocampus. These effects were accompanied by activation of the antioxidant pathway, an improved cholinergic function, and an overall decrease of oxidative stress in diabetic rats. In diabetic Balb/cA mice, oral supplementation with histidine or carnosine (0.5 and 1 g/L added into drinking water) reduced inflammatory cytokines and inhibited glucose-induced low density lipoprotein oxidation and glycation, indicating a broad spectrum of protective effects [23].

Renal protection has also been reported. In a model of diabetic nephropathy, carnosine has been shown to attenuate renal tubular damage by inhibiting ferroptosis through its antioxidant and iron-chelating properties, with nuclear factor erythroid 2-related factor 2 (Nrf2) being central to this outcome [97]. Several other studies have described the role of CNDP1-carnosine axis in the progression of diabetic complications, especially diabetic nephropathy. Increased CNDP1 activity reduces both carnosine and anserine levels, worsening renal damage. On the contrary, carnosine supplementation restores their levels, improves metabolic balance, reduces oxidative stress, and preserves the podocyte number [42],[48],[58].

Chronic carnosine supplementation has shown to decrease oxidative stress and AGEs, maintain renal and hepatic function, and enhance glycemic control in type 2 diabetes models, such as high-fat diet and STZ-induced rats [79],[81],[82]. By partially scavenging reactive aldehydes, such as methylglyoxal, oral carnosine reduced glomerular damage and albuminuria in BTBR ob/ob mice, thereby reducing AGE-induced inflammation and apoptosis [91],[97]. Notably, preliminary human trials have confirmed these advantages, demonstrating that 12-week oral supplementation lowers fasting glucose and glycate hemoglobin (HbA1c).

In addition to its positive effects on diabetes, carnosine exhibits hepatoprotective properties in various *in vivo* models of acute and chronic liver injury induced by agents such as thioacetamide (TAA), ethanol, acetaminophen, and cadmium [31],[32],[34],[35]. In these studies, carnosine, either administered intraperitoneally (10–250 mg/kg/day) or via drinking water (0.5–2 g/L), showed strong protective effects. It counteracted oxidative stress, inflammation, reduced liver tissue damage, and supported the liver's natural antioxidant defenses, suggesting its potential in alleviating, or, in the best scenario, preventing chemically induced liver injury.

In lung injury and inflammation models, such as sepsis- and bleomycin-induced lung damage, carnosine treatment was effective in reducing oxidative stress, inflammatory cytokines [including TNF-α, IL-8, transforming growth factor-beta (TGF-β)], and tissue damage, while restoring antioxidant enzyme activity. These effects were associated with modulation of key signaling pathways, such as NF-κB, and apoptosis regulation. Recent data also support its antifibrotic and antioxidant potential in cholestasis-induced pulmonary injury, reinforcing its relevance in lung-related inflammatory and fibrotic disorders [26],[71].

In the context of cardiovascular diseases, ApoE⁻/⁻ mice fed an atherogenic diet and treated with carnosine, revealed that the latter produced a reduction of atherosclerotic plaque formation along with decreased lipid peroxidation and carbonyl stress, further proving the scavenging and the anti-inflammatory properties of this dipeptide [78],[93]. Beyond metabolic and cardiovascular protection, carnosine has also shown promising antitumor properties. It inhibits tumor glycolysis, buffering extracellular acidosis, blocking vascular endothelial growth factor (VEGF)-mediated angiogenesis, and elevating oxidative stress over the threshold that malignant cells can tolerate. Although carnosine did not completely stop tumor development, mice treated with carnosine showed delayed tumor onset and slower progression compared to saline-treated controls; these effects were paralleled by a significant reduction in mitotic cells, suggesting that carnosine is effectively able to limit cancer cell proliferation *in vivo* [37].

Finally, in the ischemia-reperfusion models, carnosine preserved myocardial function and, at the same time, reduced infarct size by attenuating ROS levels and calcium overload [80],[87]. While clinical data are limited, these preclinical findings point to carnosine's capacity to modulate key pathogenic mechanisms across diseases.

## 4. Carnosine and neurodegenerative diseases

### 4.1. Cellular and molecular mechanisms of carnosine in the CNS

Carnosine neuroprotective properties are attributed to its multimodal mechanism of action, including the well-known antioxidant activity [16],[189]. *In vitro* studies have elucidated the cellular and molecular mechanisms through which carnosine exerts its effects within the CNS, particularly in the context of neurodegenerative diseases such as AD and PD [190],[191]. In AD models, carnosine has demonstrated the ability to reduce the formation of Aβ1-42 oligomers [192],[193] and to mitigate Aβ-induced oxidative stress and neuroinflammation. In particular, in BV-2 microglial cells challenged with oligomeric Aβ1-42, carnosine treatment resulted in decreased production of ROS and NO, along with a downregulation of iNOS and NADPH oxidase (Nox) enzymes [190]. Furthermore, carnosine modulated cytokine profiles by reducing pro-inflammatory IL-1β levels while enhancing anti-inflammatory cytokines such as IL-10 and transforming growth factor-beta 1 (TGF-β1). Notably, the neuroprotective effects of carnosine were blocked after inhibition of TGF-β1 signaling, underscoring the pivotal role of this pathway in mediating its protective actions [190]. Consistently, researchers further characterized the immunomodulatory profile of carnosine in BV-2 microglial cells challenged with Aβ oligomers [194]. Carnosine treatment enhanced the gene expression of macrophage inflammatory protein 2-alpha (CXCL2) and IL-10, remarking its anti-inflammatory effects. Moreover, it promoted a phagocytic phenotype by upregulating CD11b and CD68, and by restoring the fraktaline receptor (CX3CR1) expression, a chemokine receptor involved in the fractalkine signaling implicated in microglial communication and Aβ clearance. Notably, carnosine also transcriptionally increased TGF-β1 and its receptor expression, confirming the involvement of this pathway in promoting carnosine's neuroprotective response.

The ability of the dipeptide to serve as an Aβ antiaggregant has been also proved in novel formulations involving hyaluronan-carnosine conjugates [195]. Synthetic derivatives of hyaluronic acid functionalized with carnosine demonstrated a synergistic activity from the parent compounds able to inhibit the formation of amyloid-type aggregates of Aβ1-42, showing an effect directly proportional to the amount of carnosine loaded in the formulation. Moreover, the novel formulation was also able to dissolve the amyloid fibrils and to reduce Aβ-induced toxicity *in vitro* in neuroblastoma cells. Interestingly, authors speculated that one of the indirect methods used by hyaluronan-carnosine conjugates to facilitate the Aβ clearance was related to the ability to affect the early stage of Aβ enzymatic degradation mediated by the insulin-degrading enzyme (IDE). This effect seems to result from a conformational modulation of Aβ structure, rendering it more accessible to IDE-mediated proteolysis. In this context, a different research group confirmed the same concept, also highlighting the pivotal role of IDE in carnosine neuroprotection [195]. In rat mixed neuronal cultures, carnosine showed a protective behavior against Aβ1-42 oligomers-induced toxicity; interestingly, the pharmacological inhibition of IDE in the presence of 6bK, a highly selective IDE inhibitor, abolished carnosine's neuroprotective actions, confirming a causal link between IDE function and carnosine efficacy. The authors also demonstrated that the IDE activating role of carnosine was due to the increase in the oligomerization and in the cooperativity of the enzyme, which facilitates the degradation of long substrates (including Aβ peptides). Confirming the suggestions from the above-mentioned work, the results showed that carnosine is able to improve the activity of IDE by enhancing the affinity for long substrates and, in turn, the overall catalytic activity, avoiding a direct binding with the enzyme.

Recent evidence has further highlighted the antioxidant role of carnosine in the context of AD-related neuroinflammation, particularly considering the cross-talk between microglia and astrocytes, two cell types crucial for supporting neuronal functions [197]. In the study conducted on an AD model of primary mixed glial cultures, composed of both microglia and astrocytes from rat, the exposure to Aβ oligomers induced a significant increase in intracellular ROS and NO levels, while the co-treatment with carnosine markedly reduced these oxidative stress markers, preventing cytotoxic effects. In addition, single-cell analysis revealed that carnosine not only suppressed the mean levels of ROS, but also minimized cell-to-cell variability in oxidative responses, suggesting a stabilizing effect on glial reactivity [197].

In a PD context, specifically performed on GT1–7 hypothalamic neuronal cells treated with 6-hydroxydopamine (6-OHDA), a neurotoxin that induces PD-like pathology, carnosine administration attenuated cell death and suppressed the expression of integrated stress response (ISR)-related genes, including the CCAAT-enhancer-binding protein homologous protein (CHOP), the growth-arrest and DNA-damage-inducible gene 34 (GADD34), and the activating transcription factor 4 (ATF4). Additionally, carnosine inhibited ROS production and the activation of the c-Jun N-terminal kinase (JNK) pathway, suggesting a mechanism involving the suppression of oxidative stress-mediated apoptotic signaling [191]. In *in vivo* and *in vitro* rat models of PD, performed by salsolinol-induced toxicity, Zhao *et al*. demonstrated that carnosine administration restored antioxidant enzyme levels, including superoxide dismutase (SOD), catalase, and glutathione, reduced lipid peroxidation (malondialdehyde (MDA)), and decreased ROS in brain tissue and rat brain endothelial cells [155]. Histopathological analysis further confirmed that carnosine attenuated neuronal apoptosis, preserving cellular architecture. The proposed results validate the antioxidant and cytoprotective effects of carnosine in a toxin-based PD model, remarking its potential to mitigate oxidative damage to the neurovascular unit. The dysregulation of metal ions observed in PD models and the vascular type of dementia, particularly zinc, contributes significantly to oxidative stress and dopaminergic neurodegeneration. Carnosine, due to its metal-chelating capacity and antioxidant properties, has been proposed as a protective agent against zinc-induced neurotoxicity [198]. Notably, carnosine was shown to inhibit the Zn^2+^-induced expression of ER-stress-related genes, including GADD34 and CHOP [199], attenuate zinc-induced mitochondrial dysfunction, reduce intracellular ROS accumulation, and preserve dopaminergic neuron viability [200]. These findings support the hypothesis that carnosine may counteract zinc-mediated oxidative pathways implicated in the pathogenesis of PD and vascular dementia.

The neuroprotective properties of carnosine in an *in vitro* model of hemorrhagic stroke have been demonstrated [201]. The authors, by employing this model, were able to demonstrate a block of AMPA, NMDA GABA receptors' activity as a consequence of autoblood (blood clot), while an opposite effect was observed with carnosine, that when used as a pre-treatment was able to restore the activity of these key receptors. Carnosine was also able to reduce tissue swelling in olfactory cortex slices of hypertensive rats under autoblood. The protective activity of carnosine on the brain microvascular endothelial cell environment was also proved in a mouse *in vitro* model exposed to rotenone [202]. Interestingly, authors found that carnosine reduced the number of apoptotic cells and restored the decrease in mitochondrial membrane potential due to rotenone treatment, while these effects were reversed by the treatment with histamine H1 receptor antagonists (pyrilamine and diphenhydramine) and H2 receptor antagonists (cimetidine and zolatidine). These findings indicate that carnosine's ability to protect brain microvascular endothelial cells under oxidative status may be mediated by H1 and H2 receptors. The role of carnosine in the link between blood flow and ischemic stroke has been also proved in endothelial F-2 cells, in which the dipeptide showed to strongly modulate NO signaling [203]. In this *in vitro* study, carnosine dose-dependently enhanced NO production, while in contrast, its components β-alanine and L-histidine and analogue anserine (*N*-methyl-carnosine) failed to increase NO production. Notably, carnosine increased intracellular Ca²⁺ levels, essential for NO production, even in the absence of extracellular Ca²⁺. Although carnosine did not induce phosphorylation of endothelial NOS (eNOS) at Ser1177, the findings suggest that the dipeptide is able to activate eNOS via a Ca²⁺-dependent but phosphorylation-independent mechanism, involving the release from intracellular Ca²⁺ storage.

The specific activity of carnosine on astrocytes has been also widely assessed. *In vitro* studies have revealed that carnosine is able to protect astrocytes against NO-induced mitochondrial dysfunction by reducing the expression of oxidative stress-responsive genes, including poly (ADP-ribose) polymerase-1 (PARP-1) and -2 (PARP-2), thus preserving cellular integrity under stress conditions [204]. Furthermore, in primary astrocytes exposed to oxygen-glucose deprivation/recovery (a model mimicking ischemia), millimolar concentrations of carnosine reversed the expression of matrix metalloproteinase-9 (MMP-9), promoting axonal regrowth in astrocyte-neuron co-cultures [205]. These findings underline the ability of carnosine to enhance the astrocytic functions, promoting neuro-regeneration after injury. An additional *in vitro* study has also demonstrated that carnosine is available to be phagocyted by RAW 264.7 macrophagic cells, an *in vitro* model frequently used to mimic the microglia behavior, particularly under oxidative stress conditions, where its uptake can increase up to threefold [206]. This selective accumulation suggests a role for carnosine in modulating microglial oxidative responses. Indeed, carnosine treatment was reported to reduce the production of ROS in nanoparticle-exposed microglia, implicating its potential ability in counteracting environmental neuroinflammation [207]. These *in vitro* results underscore carnosine's multimodal mechanism of action in brain cells, including astrocytes and microglia, highlighting its antioxidative and anti-inflammatory capabilities that may be crucial in the context of neurodegeneration and CNS injury. Finally, carnosine has also been shown to stimulate the secretion of neurotrophins such as Brain-Derived Neurotrophic Factor (BDNF) and Nerve Growth Factor (NGF) in human glioblastoma-derived U-87 MG cells, but not in neuronal SH-SY5Y cells, suggesting a cell type-specific mechanism of action [208].

**Figure 2.**
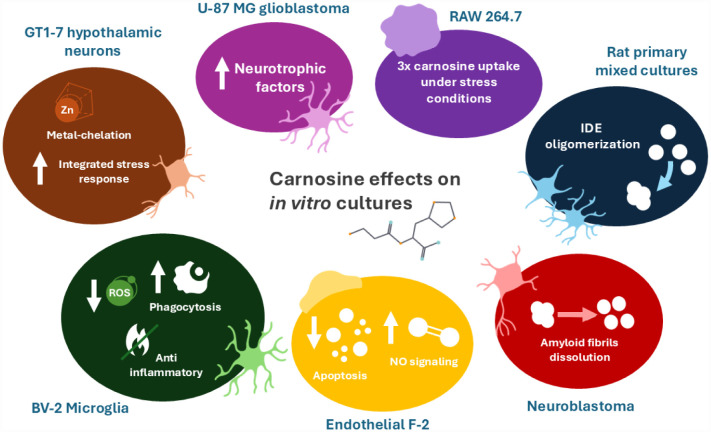
CNS *in vitro* models and molecular mechanisms modulated by carnosine. *In vitro* carnosine treatment has shown cell line-specific effects. In GT1–7 hypothalamic neurons, it enhances metal chelation and supports the integrated stress response. In U-87 MG glioblastoma cells, it increases the production of neurotrophic factors. In RAW 264.7 macrophages, carnosine uptake is tripled under stress conditions. In rat mixed cultures, it promotes insulin-degrading enzyme (IDE) oligomerization. In neuroblastoma cells, it facilitates the dissolution of Aβ oligomers. In endothelial F-2 cells, it reduces apoptosis and enhances nitric oxide (NO) production. In BV-2 microglial cells, carnosine displays anti-inflammatory activity, reduces reactive oxygen species (ROS) production, and increases phagocytosis.

**Figure 2** summarizes the *in vitro* models employed to investigate the therapeutic potential of carnosine at the CNS level.

### 4.2. Pathologies characterized by carnosine deficiency and/or impairment of carnosine-related enzyme activities

As described and clearly reported in Table 1, the therapeutic potential of carnosine has been investigated in a plethora of pathological conditions, underlining the strong consideration of this dipeptide as a possible treatment. It is also worth considering the “physiological importance” of carnosine; in fact, a well-balanced metabolism of carnosine and the maintenance of its homeostatic levels in biological fluids (e.g., blood and urine) and tissues have been reported.

The most representative deficiency in carnosine metabolism is represented by a decreased or absent activity of CNDP1 enzyme, leading to carnosinemia. The latter is a rare metabolic disorder characterized by increased levels of carnosine in urine (carnosinuria), low levels or absence of carnosinase in the blood, and, most importantly, severe neurological symptoms and developmental delays in humans [4]. From a genetic point of view, carnosinemia has an autosomal recessive pattern of inheritance, with the defective gene that has been identified to be on chromosome 18 (18q21.3). Low activity of CNDP1 has also been coupled to homocarnosinemia with increased liquor and plasma levels of the carnosine's homolog homocarnosine (a dipeptide composed by GABA and histidine) as well as urinary excretion of homocarnosine; these metabolic alterations were accompanied by moderate neuropathological symptoms [209].

With regard to abnormal carnosine levels in biological fluids, carnosinuria has been reported in children suffering from cerebro-macular degeneration, as described by Bessman and Baldwin [210] and Levenson *et al*. [211] more than 50 years ago. Fonteh *et al*. investigated free amino acid and dipeptide changes in the body fluids from AD subjects, demonstrating that carnosine levels were significantly lower in plasma of probable AD subjects compared to controls (an opposite trend was observed for L-DOPA) and linking the abnormal levels of the dipeptide to cognitive impairment measured by mini-mental state examination (MMSE) and Alzheimer's Disease Assessment Scale-cognitive subscale (ADS-cog) [212]. Of note, significantly reduced levels of carnosine compared to healthy subjects have also been coupled to mitochondrial energetic impairments in age-related macular degeneration (AMD) patients [213]. Going back to AD, in a study carried out by Balion *et al*., carnosinase activity was measured in patients with AD and mixed dementia, showing altered activities of the CNDP1 enzyme in patients with dementia [214]. Reduced serum carnosinase activity has also been observed in patients with other CNS disorders such as PD and multiple sclerosis [215].

### 4.3. AD, PD, and stroke

**Figure 3.**
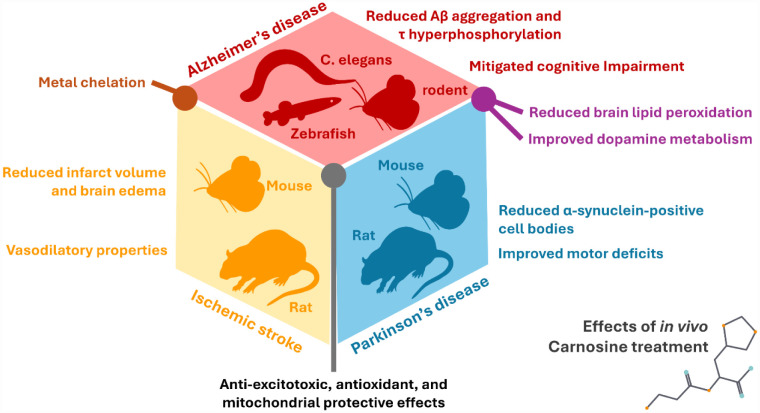
Neuroprotective potential of carnosine in cerebral ischemia, AD, and PD *in vivo* models. Rodent models have been used to demonstrate the neuroprotective potential of carnosine in cerebral ischemia and Parkinson's disease (PD). In ischemic stroke, carnosine reduces infarct volume and brain edema, chelates zinc ions, and promotes vasodilation through histamine production derived from histidine. In PD models, it decreases α-synuclein-positive cell bodies and improves motor deficits. Carnosine also reduces brain lipid peroxidation and enhances dopamine metabolism, a mechanism also observed in Alzheimer's disease (AD), where it further decreases Aβ aggregation and τ hyperphosphorylation, and improves cognitive function. In AD research, carnosine's effects have been studied not only in rodents but also in nematodes (*C. elegans*) and fish (zebrafish). Across all models, carnosine has consistently shown anti-excitotoxic, antioxidant, and mitochondrial-protective properties.

Carnosine supplementation has been reported to have beneficial effects on different experimental models of aging-related and neurodegenerative pathologies, including multiple sclerosis, depression, and post-operative cognitive dysfunction. However, in the following review sections, our interest was to examine the scientific literature on acute cerebral ischemia, AD, and PD models. Figure 3 reports the molecular pathways modulated by carnosine related to its neuroprotective potential in the context of these pathological conditions.

#### 4.3.1. Ischemic stroke

Stroke remains one of the leading causes of death globally, with ischemic stroke, caused by obstruction of blood flow, accounting for approximately 87% of all cases [216]. In acute ischemic stroke, the rapid loss of blood flow to an area of the brain results in a consistent loss of neurologic functions. Such blood decrease rapidly causes neuronal necrosis, followed by inflammatory cascade and significant oxidative stress stimulation during reperfusion. Therefore, treatments that mitigate cell damage (oxidative stress and neuroinflammation) until blood supply is restored are urgently needed, and our aim is to provide greater clarity and organization to the literature on this topic.

Carnosine has shown important neuroprotective effects in animal models of ischemia. It has been shown to significantly reduce infarct volume and brain edema following ischemic stroke. Numerous published studies have demonstrated that carnosine shows strong efficacy in ischemic stroke models [217]. In these studies, infarct volume was used as the primary outcome measure, while the neurobehavioral score was the secondary outcome. Eight animal stroke studies (454 subjects) showed that carnosine effectively reduces infarct volume, with the highest efficacy at a dose of 1000 mg/kg. The treatment was most effective when given within 6 hours of ischemia onset, but showed some benefit beyond that window. This timing is clinically relevant, as the median hospital arrival time for stroke patients is 4.3 hours [218]. This is in line with findings by Park *et al*. [139], where early intraperitoneal administration of carnosine (up to 500 mg/kg) significantly reduced infarct volume and increased antioxidant expression, emphasizing the importance of early intervention.

In rats, carnosine demonstrated strong protective effects in permanent and transient ischemic stroke models, with therapeutic frames up to 9 and 6 hours, respectively. Together with improved histological and functional outcomes, carnosine exerted anti-excitotoxic, antioxidant, and mitochondrial protective actions [126],[169]. Rats treated with carnosine, prior to inducing a transient ischemia, exhibited smaller areas of neuronal damage and a better cell survival in vulnerable regions compared to untreated controls. This effect is partly due to the reduction in oxidative and nitrosative stress during reperfusion, as well as the inhibition of astrocyte and microglia activation and the decrease in excitotoxic mediators (e.g., extracellular glutamate, which is taken up more by astrocytes because of carnosine) [123],[219]. Additional mechanistic insight comes from pMCAO models where carnosine has been shown to activate STAT3/Pim-1 pathway, upregulated Bcl-2, and reduced caspase-3, indicating its involvement in the regulation of anti-apoptotic signaling [136]. Moreover, carnosine possesses vasodilatory properties through its hydrolysis into β-alanine and L-histidine and local histamine formation, which may help improve perfusion in the penumbral regions surrounding the infarct. Noteworthy, the therapeutic window and the administration route are critical for the efficacy of carnosine. Indeed, carnosine administration (i.p.) immediately after the arterial occlusion procedure appears to be effective, while a delay in the treatment diminishes its efficacy [137],[138]. This suggests that carnosine, in a clinical setting, might be considered an acute neuroprotective treatment to be administered early during stroke/reperfusion and in combination with other drugs or therapies.

Treatment with carnosine on ischemic stroke mice, whether given as a preventive measure or within the first hour post-stroke, reduced the extent of brain lesions and improved neurological recovery. Indeed, Kim *et al*. demonstrated a neuroprotective effect for carnosine by inhibiting MMP-2 and MMP-9 activity through zinc chelation [175]. Given the crucial role of blood-brain barrier (BBB) preservation in limiting secondary injury, this aligns with recent anti-stroke strategies that increasingly focus on BBB-permeable drugs with multiple sites of action. In parallel, β-alanine, a natural precursor of carnosine, has also shown neuroprotective properties. Kopach *et al*. demonstrated that β-alanine protects brain cells under stroke-like conditions via multiple, distinct pharmacological mechanisms [220]. Given its ability to cross the BBB and its dietary availability, β-alanine also holds promise as an adjunctive therapy for stroke recovery.

#### 4.3.2. Alzheimer's disease

Numerous preclinical studies have investigated the effect of carnosine on *in vitro* or *in vivo* models of AD to determine its effect on pathological hallmarks such as amyloid aggregation, oxidative stress, and neuroinflammation.

As mentioned, one part of the scientific works had investigated the effect of carnosine by using *in vitro* models, showing how it protects against Aβ-induced toxicity by preventing amyloid aggregation and by diminishing oxidative stress and inflammation [190],[192],[193]. Carnosine acts by lowering intracellular NO and iNOS expression, modulating cytokine release, and enhancing TGF-β production. It also promotes neurotrophins expression (NGF, BDNF) [208] and simultaneously prevents their degradation by inhibiting metalloproteases.

Moving to *in vivo* studies, and in particular those involving *C. elegans*, carnosine reduced Aβ aggregation by activating the cytosolic unfolded protein response via Heat shock factor protein 1 (HSF-1) and downstream chaperones, improving proteostasis and attenuating AD-like phenotypes [221]. Many AD patients exhibit dysregulation of metal ions, such as copper and zinc, leading to accumulation of Aβ aggregation. In a *C. elegans* model of AD, copper chelation with β-alanine has been shown to reduce ROS production and to modulate the expression of oxidative stress genes, therefore increasing lifespan [222]. In a zebrafish AD model induced by exposure to okadaic acid, carnosine administration prevented cognitive and motor deficits, reduced tau hyperphosphorylation and Aβ accumulation, and preserved dopaminergic function [187]. These results highlight its multimodal neuroprotective effects, particularly in early-stage AD-like pathology. In rodent models, carnosine supplementation led to decreased Aβ accumulation, reduced lipid peroxidation, and improved mitochondrial function, resulting in enhanced cognitive performance. These effects were linked to carnosine's antioxidant and Zn²⁺-chelating properties [130]. In a rat STZ-induced model of cognitive impairment (i.c.v. injection), daily oral carnosine (100 mg/kg) supplementation, along with swimming paradigm, contributes by restoring hippocampal FNDC5/irisin expression, improving insulin signaling, and reducing Aβ and phosphorylated tau levels. These effects were paralleled by enhanced BDNF expression and improved cognitive performance. Overall, carnosine showed neuroprotective effects comparable to exercise, supporting its therapeutic potential in AD-like conditions [182]. The 3xTg-AD mouse is a widely used transgenic model of AD that carries mutations in APP (Swedish), MAPT (P301L), and PSEN1 (M146V), and is known to develop Aβ plaques and neurofibrillary tangles [223]. In 3xTg-AD mice, chronic supplementation with carnosine (10 mM in drinking water) led to a significant reduction in intraneuronal Aβ accumulation within the hippocampus and improved mitochondrial function, with a trend toward the amelioration of cognitive deficits [130].

In APP/PS1 mice, treatment with anserine, the N-methylated form of carnosine, improved spatial memory and significantly reduced neuroinflammation and pericyte degeneration, two key elements at the basis of neurovascular dysfunction. In 3xTg-AD mice fed a high-fat diet (HFD), daily treatment with carnosine (5 mg/day for 6 weeks) effectively prevented cognitive decline, with treated animals displaying memory performance comparable to controls. Although no significant differences in senile plaque load were detected across groups, carnosine markedly reversed HFD-induced microglial activation in the hippocampus and reduced receptor for advanced glycation end-product (RAGE) overexpression in cerebral blood vessels. These findings highlight carnosine's protective role against diet-induced neuroinflammation and cerebrovascular dysfunction. In parallel, anserine boosted pericyte coverage, reduced astroglial activation, and lowered IL-1β levels, suggesting a protective effect on the neurovascular unit [135]. These findings highlight the effect of anserine in counteracting cognitive decline through vascular and anti-inflammatory actions. Furthermore, in a type 2 diabetes model, carnosine improved cognitive deficits without affecting blood glucose levels or body weight. It enhanced antioxidant response, reduced lipid peroxidation, and modulated autophagy via activation of the Akt/mTOR pathway. These effects were accompanied by reduced neuronal damage and a better memory performance. Thus, carnosine appears to be effective by reducing diabetes-related cognitive decline through mechanisms independent of glycemic control [183].

#### 4.3.3. Parkinson's disease

Several researchers have assessed the potential of carnosine to treat PD, where oxidative stress and mitochondrial dysfunction play key roles in pathogenesis. Here, we present the results obtained in studies where carnosine's beneficial effects have been demonstrated in neurotoxin-induced and genetic knockout animal models of PD.

In an MPTP-induced mouse model of PD, pre-treatment with carnosine (administered via drinking water [0.5–2 g/L for 4 weeks]) significantly attenuated oxidative stress and inflammation in the striatum, thus protecting against neuronal death. This was accompanied by an improvement in motor deficits induced by MPTP, along with a reduction in brain lipid peroxidation [127]. Similarly, in a rotenone-induced rat model of PD, a nanomicellar complex of carnosine and lipoic acid administered intraperitoneally (25–50 mg/kg) reduced oxidative stress, improved antioxidant capacity, increased neuron density in the *substantia nigra*, and partially restored dopamine levels and motor activity, supporting the therapeutic potential of carnosine formulations with enhanced bioavailability [224]. In addition, carnosine has been shown to exert neuroprotective effects by enhancing the activity of antioxidant enzymes such as glutathione peroxidase (GPX) and superoxide dismutase (SOD) while reducing pro-inflammatory markers, including IL-6, TNF-α, and iNOS. Its combined antioxidant and anti-inflammatory effects also improved dopamine metabolism, helping to protect dopaminergic neurons from degeneration and cell death.

According to Zhao and collaborators [155], carnosine treatment in salsolinol-induced rat models demonstrated significant neuroprotective effects by not only normalizing antioxidant enzyme levels and reducing lipid peroxidation markers, but also by substantially decreasing mitochondria-derived ROS production in endothelial cells while preserving cellular architecture integrity in brain tissue. These findings suggest that carnosine's protective mechanism extends beyond conventional antioxidant activity to encompass mitochondrial function preservation, an effect consistent with broader preclinical research demonstrating carnosine's multimodal actions, including antioxidant, anti-inflammatory, and anti-aggregate properties across neurodegenerative disease models, including PD, thereby supporting its potential as a disease-modifying agent [189].

Beyond neurotoxicity-induced models, carnosine administration has also been tested on transgenic models of PD. In a study conducted on mice overexpressing human α-synuclein (Thy1-aSyn model [167]), two carnosine administration routes were compared: the intranasal route versus the oral route. Transcriptomic analysis revealed that α-synuclein overexpression was primarily associated with impaired ribosomal biogenesis and compromised mitochondrial function. Notably, after two months of treatment, mice receiving carnosine treatment intranasally exhibited significantly amelioration in gene expression profiles and mitochondrial function compared to oral treatment, suggesting that this therapeutic approach effectively targets key molecular pathways disrupted in PD pathogenesis through enhanced brain bioavailability.

A subsequent study demonstrated that intranasal carnosine administration (2.0 or 4.0 mg/day for 8 weeks) produced dose-dependent improvements in motor function in Thy1-aSyn mice [176]. Complementary results on the same Thy1-aSyn model have been obtained in Bermúdez *et al*. with intranasal carnosine treatment (2 mg/day for 2 months) [167],[168]. Moreover, researchers have showed preserved gait performance and reduced α-synuclein immunoreactivity in the olfactory epithelium, further supporting the enhanced efficacy of nasal administration and its ability to limit pathological protein deposition [168]. Furthermore, carnosine treatment significantly reduced α-synuclein-positive cell bodies in the *substantia nigra pars compacta* to levels comparable with vehicle-treated wild-type mice, indicating that carnosine reduces pathological α-synuclein accumulation in motor-related brain regions, offering mechanistic support for its therapeutic potential in PD progression. Overall, in PD, carnosine appears to maintain redox homeostasis in dopaminergic cells and prevent the formation of toxic α-synuclein oligomers. In a 6-OHDA-induced PD model, pre-treatment with carnosine (250 mg/kg i.p.) significantly attenuated motor symptoms, apoptosis, and oxidative damage, confirming its early neuroprotective action in dopaminergic degeneration [148].

Beyond AD and PD, carnosine has been evaluated in other neurological contexts, showing beneficial effects in models of depression (enhancing hippocampal neurogenesis in chronically stressed rats) [119], seizure [117],[122],[164], autism [181], and in cognitive dysfunction (where it appears to mitigate glial inflammation [186]). This broad spectrum of actions put carnosine as a homeostatic modulator of the neuronal microenvironment that probably acts by rocketing existing neuroprotective pathways than the activation of new mechanisms.

## 5. *In vivo* research on carnosine: What was achieved and what is missing

Despite the very promising findings reported above, it is very challenging to compare results across so many studies due to the great heterogeneity in terms of experimental approaches used, including different species (from worms to different rodent strains), a wide range of dosages along with very different treatment durations, and multiple delivery routes. Additionally, one of the most urgent needs, enabling us to better comprehend the enormous therapeutic potential of carnosine, is represented by carrying out pharmacokinetics studies, connecting the dose administered to the one arriving at the site of action and able to provide the benefit observed. In this regard, a very recent and informative paper was published by Ali *et al*. [225]. The authors employed proton magnetic resonance imaging (MRI) spectroscopy to evaluate the effect of oral dosing on brain carnosine concentrations in humans while investigating safety and tolerability, providing information on dosing interventions in future clinical trials. Similar studies should be re-proposed *in vivo* by employing the same models, then enabling us to better understand the efficacy of this pleotropic endogenous molecule. Moving in this direction, in collaboration with Prof. Lunte's group at the University of Kansas, we carried out pharmacokinetic studies on rats in which carnosine bioavailability at the brain level (e.g., ventral striatum), following the i.p. administration of 250 mg/kg of carnosine, was measured through microdialysis, a preferred sampling method for continuous monitoring of biomolecules in the extracellular space of various organs such as the brain [226]. Our preliminary results suggest that a single i.p. administration ensures a quick increase of carnosine at the brain level (i.e., 10 minutes after the injection), increasing its basal concentration up to 4 times compared to the basal level, with the increase being stable for about 40 minutes. Further studies are ongoing.

The inhibition of carnosinase activity has emerged as a promising strategy to prolong carnosine's half-life and increase its therapeutic effect. Bestatin is a dipeptide containing an unusual β-amino acid that makes it resistant to hydrolysis by carnosinase and rapidly forms a stable enzyme–inhibitor complex. This interaction effectively blocks carnosinase activity, with a higher affinity for CNDP2 than for CNDP1 [227], thereby preserving circulating histidine-containing dipeptides and improving their bioavailability *in vivo* [228]. Similarly, carnostatin (SAN9812) has been identified as the first selective and effective inhibitor of CNDP1 [229]. It acts as a competitive inhibitor, preventing the rapid hydrolysis of carnosine, and thereby enhancing its stability and availability in the body, which in turn increases its antioxidant and anti-glycating effects [230]. Its action has been also validated in transgenic mice expressing the human CNDP1 gene, where the co-administration with carnosine resulted in a sustained increase of circulating carnosine without adverse effects on the central nervous system. Beyond these, additional approaches have been proposed, such as the carnosinase-resistant D-carnosine pro-drug, D-carnosine-octylester [231], that proved to be successful in attenuating atherosclerosis and renal disease induced by a western diet or streptozotocin-induced diabetes in the ApoE-null mice [47],[63]. Another approach that has been explored is the use of carnosinol, a (2S)-2-(3-amino propanoylamino)-3-(1H-imidazol-5-yl)propanol (FL–926–16), a new carnosinase-resistant L-carnosine derivative that has shown to counteract diabetic nephropathy [76]. Together, this evidence strengthens the concept that combining carnosine supplementation with pharmacological inhibition of its degrading enzymes could represent a powerful and effective strategy to fully extend the complete therapeutic potential of this dipeptide.

Last but not least, it is important to underline the use of carnosine in combination with anserine, a natural derivative of carnosine usually adopted because it is not cleaved by human carnosinases. This “protocol”, which is very often considered and used at the clinical level [232]–[238], is almost completely missing in preclinical studies, suggesting that, like the studies mentioned, considering the supplementation of carnosine in combination with anserine may lead to better results, providing a better alignment between preclinical and clinical studies.

## 6. Future perspectives and concluding remarks

Since its discovery, thousands of research studies have been carried out to investigate the role, function, and biological activities of carnosine under physiological and pathological conditions, illustrating the substantial attention that this molecule has garnered within the scientific community. With regard to *in vivo* studies, both the importance of maintaining carnosine physiological levels as well as its multimodal mechanism of action in numerous systemic and neurodegenerative disorders have been well demonstrated. Despite the enormous therapeutic potential, our focus of this review was to critically highlight the strengths and weaknesses of carnosine, as in the case of the significant heterogeneity regarding administration route, dosage, treatment, and animal model selected. More translational studies are needed to better understand the literature, which will enable us to accurately plan future and well-defined clinical studies.

## Use of AI tools declaration

The authors declare they have not used Artificial Intelligence (AI) tools in the creation of this article.
